# Unusually Warm Summer Temperatures Exacerbate Population and Plant Level Response of *Posidonia oceanica* to Anthropogenic Nutrient Stress

**DOI:** 10.3389/fpls.2021.662682

**Published:** 2021-07-05

**Authors:** Stephanie B. Helber, Gabriele Procaccini, E. Fay Belshe, Alex Santillan-Sarmiento, Ulisse Cardini, Stefanie Bröhl, Michael Schmid, Hauke Reuter, Mirta Teichberg

**Affiliations:** ^1^Leibniz Centre for Tropical Marine Research (ZMT) GmbH, Bremen, Germany; ^2^Institute for Chemistry and Biology of the Marine Environment, University of Oldenburg, Wilhelmshaven, Germany; ^3^Department of Integrative Marine Ecology, Stazione Zoologica Anton Dohrn, Naples, Italy; ^4^Faculty of Engineering, National University of Chimborazo, Riobamba, Ecuador; ^5^Faculty of Biology and Chemistry, University of Bremen, Bremen, Germany

**Keywords:** Mediterranean, global warming, nutrients, multiple stressors, warning indicators, seagrass degradation

## Abstract

*Posidonia oceanica* is a key foundation species in the Mediterranean providing valuable ecosystem services. However, this species is particularly vulnerable towards high coastal nutrient inputs and the rising frequency of intense summer heat waves, but their combined effect *in situ* has received little attention so far. Here, we investigated the effects of *in situ* nutrient addition during an unusually warm summer over a 4-month period, comparing different morphological, physiological and biochemical population metrics of seagrass meadows growing in protected areas (Ischia) with meadows already exposed to significant anthropogenic pressure (Baia – Gulf of Pozzuoli). Our study highlights that the effects of warmer than usual summer temperatures on the population level of seagrass meadows can be exacerbated if the plants are already exposed to higher anthropogenic pressures. Morphological and population level indicators mainly changed over time, possibly impacted by season and the warmer temperatures, and displayed more pronounced reductions in seagrasses from impacted sites. The additional nutrient supply had even more deleterious effects, as shown by a decrease in approximately 67% in cover in fertilized plots at high impacted sites and 33% at low impacted sites. Moreover, while rhizome starch concentration showed a seasonal increase in plants from low impacted sites it displayed a trend of a 27% decrease in fertilized plots of the high impacted sites. Epiphyte biomass was approximately four-fold higher on leaves of plants growing in impacted sites and even doubled with the additional nutrient input. Predicting and anticipating stress in *P. oceanica* is of crucial importance for conservation and management efforts, given the limited colonizing and reproductive ability and extremely slow growth of this ecosystem engineer. Our results suggest that monitoring efforts should focus especially on leaf area index (LAI), carbohydrate concentrations in the rhizomes, and epiphyte cover on leaves as indicators of the onset of stress in *Posidonia oceanica*, which can be used by decision makers to take appropriate measures before damage to the ecosystem becomes irreversible, minimize future human interference and strengthen the resilience of these important ecosystems.

## Introduction

Coastal vegetated ecosystems are facing multiple local and global anthropogenic stressors that affect their health and their associated ecosystem services (Orth et al., 2006; Duarte et al., 2008; Hoegh-Guldberg and Bruno, 2010). Seagrasses are one of the most valuable marine ecosystems on Earth (Costanza et al., 2014). These marine ecosystems provide a range of ecosystem services including the provision of nursery habitats, filtration of nutrients, provision of food resources and habitat for herbivores as well as their ability to sequester large amounts of carbon aiding in climate change mitigation (de la Torre-Castro and Rönnbäck, 2004; Fourqurean et al., 2012; Ondiviela et al., 2014; Bakker et al., 2016). However, they are also among the marine habitats that are experiencing the most steep decline rates, estimated at 7% year^–1^ (Waycott et al., 2009) even in relatively pristine areas (Marbà and Duarte, 2010; Arias-Ortiz et al., 2018). As a result, almost 14% of all seagrass species are endangered and 29% of the world’s seagrass meadows have been lost (Waycott et al., 2009; Short et al., 2011).

Eutrophication on a local scale and climate change on a global scale are the most prominent threats to seagrass ecosystems (Burkholder et al., 2007; Halpern et al., 2007; Marín-Guirao et al., 2018).

The wide distribution of seagrasses along coastal urbanized areas make them particularly vulnerable to anthropogenic nutrient inputs, such as those from aquaculture farms as well as from sewage, industrial or agricultural runoff (Laubier, 2005). In particular, anthropogenic-derived nitrogen in the environment increased by almost 10-fold since 1850 as a consequence of the rising demand for reactive nitrogen in energy production, coastal aquaculture and agriculture (Galloway et al., 2014).

Excess nutrient loading promotes the proliferation of fast-growing species, including phytoplankton, macroalgae and epiphytic algae which ultimately limit light availability to the leaves of seagrasses through shading or overgrowth (Cardoso et al., 2004; Burkholder et al., 2007). While moderate increases in nutrient concentrations stimulate the production and growth of seagrasses (Short et al., 1990; Alcoverro et al., 1997; Udy et al., 1999), large amounts of nitrogen are lethal for the plants because they are unable to down-regulate nitrogen uptake (Rare, 1990; Touchette and Burkholder, 2000). Plant productivity and survival can be impacted by direct ammonium toxicity as well as by the depletion of plant carbon reserves caused by the increased energy demand for rapid ammonium assimilation (Burkholder et al., 1992; Van Katwijk et al., 1997; Invers et al., 2004). Additionally, high nutrient availability results in increased leaf nutritional quality, which stimulates seagrass grazing (Prado et al., 2010; Ravaglioli et al., 2017). A further consequence of eutrophication and organic matter enrichment to the sediment is the stimulation of bacterial respiration leading to hypoxic or anoxic sediments (Frederiksen et al., 2007; Marbà et al., 2007), which can negatively affect respiration, growth and nutrient uptake of seagrass roots (Hemminga, 1998; Pérez et al., 2007). More importantly, anoxic conditions stimulate sulfate reduction and the consequent accumulation of sulfide, a strong phytotoxin (Holmer and Bondgaard, 2001; Ontoria et al., 2019), limiting seagrass survival and expansion (Calleja et al., 2007; Frederiksen et al., 2007; Govers et al., 2014a).

Global warming is another significant threat that can lead to seagrass loss (Short et al., 2016; O’Hare et al., 2018), particularly given that the frequency and magnitude of local heat waves is expected to intensify as a consequence of climate change (Meehl et al., 2005; Oliver, 2019). Indeed, high mortality rates have been reported for temperate seagrass species after extreme temperature events, e.g., for *Zostera marina* in Chesapeake Bay, Virginia, for the period of 2004–2011 (Moore et al., 2014) and *Amphibolis antarctica* following the 2010/2011 heat wave in Shark Bay, Western Australia (Thomson et al., 2015).

The Mediterranean Sea ranks among the fastest-warming ocean regions, showing rates of seawater warming double or triple of those in the global ocean (Vargas-Yáñez et al., 2008). Summer surface temperatures of the Mediterranean have already increased by 1.15°C within the last three decades, with mean global sea-surface temperatures predicted to rise another 3–4°C by the end of this century (Levitus et al., 2001; Meehl et al., 2005; Marbà et al., 2015). An increasing occurrence of heat wave events have also been documented for the Mediterranean in recent years (e.g.1994, 2003, and 2009) (Coma et al., 2009), and die-offs were reported for *P. oceanica* in 2003 and 2006 (Marbà and Duarte, 2010). Seagrass shoot losses as well as impacts on the photosynthetic rates of adult *P. oceanica* plants and their seedlings are expected to occur when seawater temperature reaches 27°C for several days or weeks (Marbà and Duarte, 2010; Guerrero-Meseguer et al., 2017). Die-offs of *P. oceanica* meadows reveal the vulnerability of this species to intense warming events (Marbà and Duarte, 2010; Jordà et al., 2012) and can potentially lead to ecosystem regime shifts. *P. oceanica* is an extremely slow growing plant with shoots living for several decades and limited sexual reproduction (Marbà et al., 1996; Marbà et al., 2004; Díaz-Almela et al., 2009). Therefore, *P. oceanica* meadows need a long time to recover from these mortality events (Marbà and Duarte, 2010), and early successional seagrass species, invasive species, or macroalgae can colonize the free space if given the chance (McGlathery, 2001; Nowicki et al., 2017; Beca-Carretero et al., 2020).

Global warming and eutrophication rarely occur in isolation in the environment and these stressors may interact additively, synergistically, or antagonistically (Todgham and Stillman, 2013; Egea et al., 2018; Mvungi and Pillay, 2019). Thus, it is of highest importance to understand the response of seagrass species to combined stressors in their environment, to be able to give sound guidelines for adequately managing and restoring these endangered ecosystems.

Multiple stressors can have complex and unforeseen impacts on seagrass species and the few studies performed so far reported controversial outcomes. Additive effects for ocean warming and nutrient enrichment were reported for *Cymodocea nodosa* in the Mediterranean (Ontoria et al., 2019) and *Zostera marina* in the North Sea (Moreno-Marín et al., 2018), while only limited additive effects of those stressors were found in *Zostera capensis* from South Africa (Mvungi and Pillay, 2019) and even antagonistic effects for *P. oceanica* (Pazzaglia et al., 2020) as well as limited and antagonistic effects in three tropical seagrass species (Viana et al., 2020). Moreover, limited or no interaction between nutrient and temperature stress have been observed on seagrass seedlings of the tropical species *Enhalus acoroides* (Artika et al., 2020).

These studies make clear that predicting the impact on seagrass of one stressor in isolation could grossly under- or overestimate the combined effects that seagrass ecosystems will be experiencing in their environment. Moreover, other factors such as herbivory (Bakker et al., 2016) or mechanical stress through storm events (Oprandi et al., 2020) could worsen the effects of anthropogenic pressures on seagrasses, and these factors are not taken into consideration when individual seagrass plants are studied in mesocosm experiments. On the other hand, the whole seagrass ecosystem may be able to buffer negative effects that have been observed by single plants in mesocosm settings, or possibly, unforeseen biological interactions might emerge that generate positive or negative feedbacks within the system (Burnell et al., 2013).

We undertook this study to explore the combined effects of seasonal warming and nutrient enrichment in the water column and in the sediment on *P. oceanica* meadows in their natural environment. This study is part I of a parallel study that assesses the combined effects of temperature and nutrient stress on *H. stipulacea*, a small- bodied, fast- growing seagrass species (Helber et al., unpublished). In these combined studies, we aimed to compare these two species to expand our knowledge on the influence of growth strategy on the response of seagrasses under future eutrophication and climate scenarios that might lead to competitive interactions in areas where their distribution range overlaps. Our overarching aim was to identify warning indicators for the onset of stress in these seagrass species, including individual plant trait responses and population or community-level changes, to alert decision makers and provide an opportunity to take appropriate measures before irreparable damage to the ecosystem, such as seagrass die-off or catastrophic ecosystem regime shifts, can occur.

In part I, we hypothesized that maximum summer temperatures and additional nutrient enrichment will have more detrimental effects on *P. oceanica* meadows that are already heavily exposed to anthropogenic pressures. In contrast, we expect, that small- bodied, fast- growing seagrass species are able to adapt more quickly through especially morphological but also biochemical changes, and therefore might even benefit from the additional supply of nutrients. This is further tested in Part II of this study (Helber et al., unpublished). Moreover, we expect that in both species the combined effects of high temperatures and fertilization will be larger than the effects of one stressor alone.

## Materials and Methods

### Study Site and Experimental Design

**FIGURE 1:**
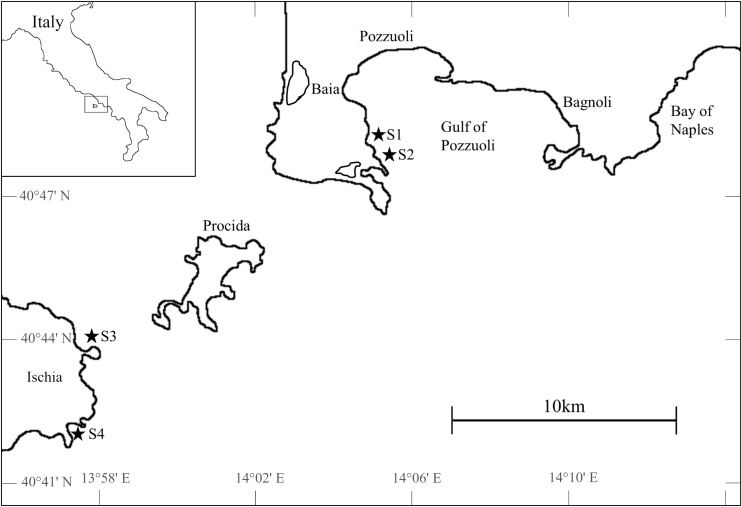
Study sites in the urbanized Gulf of Pozzuoli (S1, S2) and in the Marine Protected Area “Regno di Nettuno” off the island of Ischia (S3, S4).

We compared the response of *P. oceanica* populations to thermal and nutrient stress at two locations exposed to significant anthropogenic nutrient load vs. in a Marine Protected Area subjected to relatively low anthropogenic pressures along the western Mediterranean Sea, off the coast of Naples, Italy (Figure 1). Four sites were chosen after verifying low within-meadow heterogeneity that would allow us to provide a good assessment of meadow status and to detect changes during the experiment.

The two impacted sites are located in the Gulf of Pozzuoli close to the city of Baia (S1: 40.7986°N, 14.0856°E and S2: 40.7961°N, 14.0865°E). This area is heavily influenced by anthropogenic activities, with sediments containing high levels of organic contaminants and pollutants (Celia Magno et al., 2012; Tornero and Ribera d’Alcalà, 2014). Additionally, this area is impacted by dense urban settlements, discharge of sewage without appropriate depuration, and intense maritime traffic as well as mussel aquaculture (Tornero and Ribera d’Alcalà, 2014; Appolloni et al., 2018). Some of the mussel aquaculture facilities rearing *Mytilus galloprovincialis* are located close to our two experimental sites with a distance of 500 m from the coast, covering an area of about 257 m^2^ (Galletti et al., 2017).

Experimental sites S3 (40.7354°N, 13.9646°E) and S4 (40.7063°N, 13.9567°E) were located off the island of Ischia, which is a marine protected area since 2007 (Chiarore et al., 2019). However, Ischia attracts more than four million tourists per year with the majority visiting the island between May and September (Tomicic et al., 2001; Carlino et al., 2011). During this time, the number of people is fourfold higher than in winter, with the outdated wastewater system representing the major source of pollution (Tomicic et al., 2001). *P. oceanica* at the sites in Ischia is found in dense and continuous meadows that grow on a “matte,” a dense mixture of rhizomes, roots and accumulated sediment (Boudouresque et al., 2016), while meadows in the Gulf of Pozzuoli do not possess the same vertical extent, growing directly on sediment (Supplementary Figure 1).

Six circular plots with a diameter of 2 m were established at each of the four sites, at least 10 m apart from each other. Plots were set up at water depths between 7.4 and 12.0 m in seagrass meadows around Ischia and at 6.6–7.9 m water depth in meadows around Baia. Three plots were used as control, while the other three were enriched with slow release fertilizer pellets (Osmocote^®^ Pro: 19% N – 3.9% P – 8.3% K, ICL Specialty Fertilizers). Nitrogenous compounds were composed of nitrate (6.3%), ammonia (8.2%), and urea (4.5%). The nutrient treatment level was randomly assigned for each plot. Fertilizer pellets were filled in nine 0.5 m long punctured PVC tubes, resulting in an addition of approximately 1170 g of fertilizer per plot. PVC tubes were pushed approximately 20 cm into the sediment, delivering nutrients to the below-ground and above-ground tissues, thereby simulating the effects of long-term cultural eutrophication. From each plot, we measured a number of target variables in June and September 2019 for assessing different levels of biological organization, ranging from biochemical and morphological individual plant traits, to community level metrics as outlined below.

### Shoot Density and Percent Cover

Shoot density and percentage cover were assessed at the start of the experiment in June 2019 during the seagrass growth season and right after the maximum summer temperatures in September 2019. Shoot density (number of shoots per m^–2^) was measured by counting the number of shoots in triplicate 0.25 m^2^ quadrats randomly placed within each plot. Meadow cover (as a percentage) was quantified by visually estimating the percentage of substrate occupied by *P. oceanica* shoots within one randomly placed 1 m^2^ quadrat in each plot. To assess percent cover, the quadrat was positioned on the substratum, and seagrass leaves were moved aside to estimate the portion of underlying floor covered by shoots vs. by bare substratum.

### Seagrass Collection

From each plot, nine shoots (when possible, orthotrophic) were randomly collected at different locations within the plot to reduce the likelihood that ramets were sampled from connected horizontal rhizomes (Rico-Raimondino, 1995). Shoot collections were done at the beginning and the end of the experiment. Three shoots were used to measure morphological parameters and epibiont biomass, while the other three shoots were used to quantify C and N nutrient content and δ^13^C and δ^15^N in leaves and rhizomes and the remaining three shoots to measure total non-structural carbohydrate reserves, such as starch and sugars in leaves and rhizomes.

### Carbon, Nitrogen, and Phosphorous Tissue Content and Natural Stable Isotopes

Samples for total carbon (C), nitrogen (N), and phosphorous (P) content and δ^13^C and δ^15^N isotope analyses were collected from the second- and third-rank leaves and rhizome tissue of three randomly selected shoots per plot. Epiphytes were gently scraped off the leaves before freezing them at –20°C. Before analysis, the leaf and rhizome samples were freeze-dried for 48 h. The dried leaf and rhizome tissues were ground and total C and N content was measured using a Euro EA 3000 elemental analyser (EuroVector). Acetanilid 5 (Hexatech) was used as standard. Repeated measurements of internal standards with known C and N concentrations (Low Soil Standard OAS 5; IVA) ensured measurement accuracy. Carbon and nitrogen content were expressed as a percentage of dry weight and the values used to calculate the C:N ratios.

Percent tissue phosphorus was analyzed using the wet alkaline persulphate digestion technique method on a TECAN M200Pro plate reader after Hansen and Koroleff (2009). Fichtennadel (1.69 mg P g^–1^) and SRM1515 Apple leave (1.59 mg P g^–1^) were used as standard reference materials. Repeated measurements of these reference materials ensured measurement accuracy. The coefficient of variation was always < 3.4%.

Stable isotopic ratios of carbon (^13^C/^12^C) and nitrogen (^15^N/^14^N) in samples were analyzed using a Finnigan Delta Plus mass spectrometer coupled with a Flash EA 1112 elemental analyzer. Results of isotopic composition in samples are expressed as following:

$$\delta X\left(‰\right)=\left[\left(R_{sample}/R_{reference}\right)-1\right]\times 1000,$$

where X is either ^15^N or ^13^C, and R is the ratio of ^15^N/^14^N for nitrogen and ^13^C/^12^C for carbon. Reference materials IAEA N1 and N2 (nitrogen) and USGS 24 and NBS22 (carbon) from the International Atomic Energy Agency were used for calibration. The precision of the measurements was <0.06‰ for both carbon and nitrogen. All δ^13^C and δ^15^N values were normalized to the internal standards of wheat flour (carbon [δ^13^C]: – 27.21‰; nitrogen [δ^15^N]: 2.85‰) and high organic sediment (carbon [δ^13^C]: – 26.07‰; nitrogen [δ^15^N]: 4.4‰).

### Morphological Parameters

The number of leaves was counted from three shoots that were randomly sampled within each plot in June and September 2019. The following parameters were measured: (i) number of leaves, (ii) length of all leaves and (iii) maximum leaf width. Subsequently, leaf area and leaf area per shoot as well as leaf area index (LAI) were determined. LAI was calculated as the product of leaf surface area per shoot and density of shoots per m^2^.

### Epiphyte Cover

Epiphytes were scraped off leaves from each shoot using a scalpel blade and frozen at –20°C in falcon tubes until further processing at the ZMT in Germany. In the laboratory, epiphytes were removed with distilled water from the falcon tubes and dried to a constant weight (60°C, 48 h) to determine their biomass [mg (DW) shoot^–1^]. Epiphyte biomass was normalized to the leaf surface area of its shoot to quantify epiphyte load [mg (DW) cm^–2^].

### Carbohydrate Reserves

Triplicate subsamples of the rhizome and leaf samples were pooled and freeze-dried for 72 h. Subsequently, samples were ground with mortar and pestle. The dried powder (0.02–0.03 g) was suspended in 1.5 mL Milli-Q water and soluble sugars were extracted from the ground dry tissues by vortexing and shaking for 15 min. Samples were centrifuged (13000 rpm, 5 min) and the supernatant was used for soluble sugar determination via the Anthrone Assay (Viles and Silverman, 1949). Starch contents (total non-structural carbohydrates) in the remaining pellet were boiled in Milli-Q (10 min at 100°C) to gelatinize the starch. Subsequently, samples were hydrolysed by the enzyme alpha- amylase (80 min at 80°C). The supernatant containing oligosaccharides and/or glucose broken down by the enzyme was collected and, by boiling the sample material under acidic conditions (addition of 96% H_2_SO_4_) for 1.5 h at 100°C, the remaining polysaccharides were broken down to glucose molecules. Starch and sugar contents in the extracts were determined spectrophotometrically (620 nm) using an anthrone-sulphuric acid assay with a F200- Pro TECAN plate reader. Carbohydrate concentrations were quantified as sucrose equivalent using sucrose calibration curves (Standard sucrose 99%, from Sigma Aldrich). Samples of cellulose, glucose, and starch were used as reference samples.

### Environmental Sampling

Approximately 30 mL water samples were taken at around 10 m depth in Ischia and around 7 m depth in Baia, above the seagrass canopy of each plot on each site for water column nutrient analysis. Additionally, porewater samples were taken by placing a syringe 5 cm deep in the sediment and carefully drawing out water from interstitial spaces of the sediment to determine nutrient concentrations. Porewater samples were not able to be measured in Baia at the first fieldtrip due to sediment characteristics- dense silty soil that blocked our porewater sampling lancets. For the second fieldtrip we used Metalplex hypodermic syringes. Water samples were taken with cleaned plastic syringes and immediately filtered using sterile syringe filters (LABSOLUTE^®^; cellulose acetate; 0.45 μm pore- size) into HDPE vials and stored on ice. HDPE vials were pre-rinsed twice with sample water. Samples were immediately frozen at –20°C upon arrival in the laboratory on the same day until further analyses. Nutrient measurements were performed spectrophotometrically with a TECAN plate reader (Infinite 200 pro Microplate reader; Switzerland) according to Laskov et al. (2007). The detection limits were 0.08, 0.32, 0.7, and 0.022 μM for NO_2_^–^, NO_*x*_ (NO_3_^–^ and NO_2_^–^), NH_4_^+^, PO_4_^–^, respectively. The NO_*x*_ (NO_3_^–^ and NO_2_^–^) and NH_4_^+^concentrations sum up to dissolved inorganic nitrogen (DIN). The coefficient of variation was always <3.4%.

The water quality of the different sites was assessed by taking additional water samples on top of the seagrass canopy with 5 × 5 L seawater pre-rinsed HDPE containers. After collection, containers were immediately stored in the dark on ice. A defined volume of water was vacuum- filtered onto pre- combusted (5 h, 450°C) Whatmann^®^ GF/F filters. Filters for chlorophyll *a* (Chl-*a*) analysis were immediately stored at –80°C, filters for organic carbon (C_*org*_) and C and N content were frozen at –20°C until further analysis at the Leibniz Centre for Tropical Marine Research. Filters for C_*org*_, C and N content were divided in four equal pieces and one piece was used for analysis. For Chl-*a* analysis, filters were analyzed and extracted in 80°C hot Ethanol after Welshmeyer (1994). The supernatant was subsequently transferred into small vials and Chl-*a* concentrations were determined with a TD10AU-Fluorometer (Turner Designs). The detection limit of this method is 0.002 μg l^–1^. Some water parameters of the first fieldtrip could not be measured because filters got lost during the transport.

Salinity and pH were measured at the same time the water samples were taken at each site with a multi parameter probe (WTW Multiprobe). Moreover, temperature loggers (HOBO Water Temp Pro v2) were fixed to the main pole of plot 1 and plot 6 in the seagrass meadow to record the water temperatures hourly during the entire duration of the experiment.

### Statistics

Statistical analyses were performed in R version 3.5.3 (R Core Team, 2019). For the statistical analysis, we combined data for Site 1 and 2 together as high impacted condition and Site 3 and 4 together as low impacted condition. Although, there were site- specific differences, we aimed to focus on large- scale patterns and therefore only distinguished between the environmental history of the two locations. Prior to analysis, we subset our data to values that have been collected in June 2019 (start of the experiment) and September 2019 (end of the experiment). The fertilization was only started in June and thus there was no nutrient treatment effect in June. We used two linear mixed effects models. One to determine if there were seasonal effects over the time of the experiment (1). For this analysis, we only looked for changes in the control plots over time and included a random intercept for site. The second linear mixed effects model was performed to look at differences between the conditions of the sites (high or low impacted) in June (2) and September (3), the effect of treatment- differences between fertilized and control plots (4) as well as their possible interactions (5). We performed both linear mixed effects models using the lme4 function in R (Bates et al., 2015). As fixed effects, we considered condition (high or low impacted), treatment (fertilization and control) and the interaction of condition and treatment. We added a random intercept of site to our linear mixed effects models to account for the variability in sites within areas of different conditions (high or low impacted). Differences in shoot densities among the sites were determined with a generalized linear mixed-effects model (GLMM), specifically a negative binomial model (link = log) because the data were counts and found to be over-dispersed (Zuur et al., 2009). The GLMM was fitted using the MASS package (Venables and Ripley, 2002). All models were validated visually with plots of model residuals (fitted values vs. absolute residuals (homogeneity of variance), a qqplot comparing the distribution of the standardized residuals to the normal distribution (normality), and a lag plot of the raw residuals vs. the previous residual (independence; Zuur et al., 2009). The significance of each independent variable (or interaction) in each model was assessed using the likelihood ratio (LR) test by comparing models with the variable of interest against the null or reduced model (Winter, 2013). To bolster the results found by LR test the models that best predicted the changes in seagrass indicators were identified by minimum Akaike Information Criterium with correction for small sample sizes (AICc), model ranking and weighing (Burnham et al., 2011; Symonds and Moussalli, 2011). Models with the variable of interest (or interaction) were compared to the null (or reduced) model and a model was considered superior model if it had the lowest AICc units (determining the model strength) as well as the highest Akaike weight (AICcWt) which defines the weight of evidence of each model relative to the null or reduced model (Arnold, 2010). All graphics were produced with the ggplot package (Wickham, 2016).

Water parameters were also combined after condition- low (Ischia) and high (Baia) impacted sites. Data were analyzed with the Kruskal–Wallis rank sum test function in R and subsequently a paired t-test with holms correction was performed.

## Results

### Environmental Variables

Average daily temperatures above 27°C were recorded for 9 days at Site 1, for 20 days at Site 2, for 7 days at Site 3 and for 15 days at Site 4 between mid-June and September 2019 (Supplementary Figure 2). Temperatures were recorded at 7.4–12.0 m water depth in seagrass meadows around Ischia and at 6.6–7.9 m water depth in meadows around Baia. Maximum temperatures recorded during this time were 28.16°C for Site 1 and 28.26°C for Site 2 in Baia (Gulf of Pozzuoli) and 27.96°C for both sites in meadows around Ischia.

There were no significant differences in nutrient concentrations in the water column and porewater between the sites, even though DIN and NH_4_^+^ seemed to be higher in the porewater at Baia (Supplementary Table 1).

Water parameters differed significantly between locations that are exposed to different levels of anthropogenic stressors. High impacted sites in Baia (Site 1 + 2) had higher concentrations of organic carbon (Corg), C and N content as well as chla contents in the water column (Supplementary Tables 1, 2). CN ratios did not differ significantly between sites in Baia and Ischia. Values for organic carbon (Corg), C and N content were always under the limit of detection for Site 3 in Ischia.

### Changes Between Seasons (June 2019 and September 2019) and With Fertilization

#### Percentage Cover and Shoot Density

**FIGURE 2:**
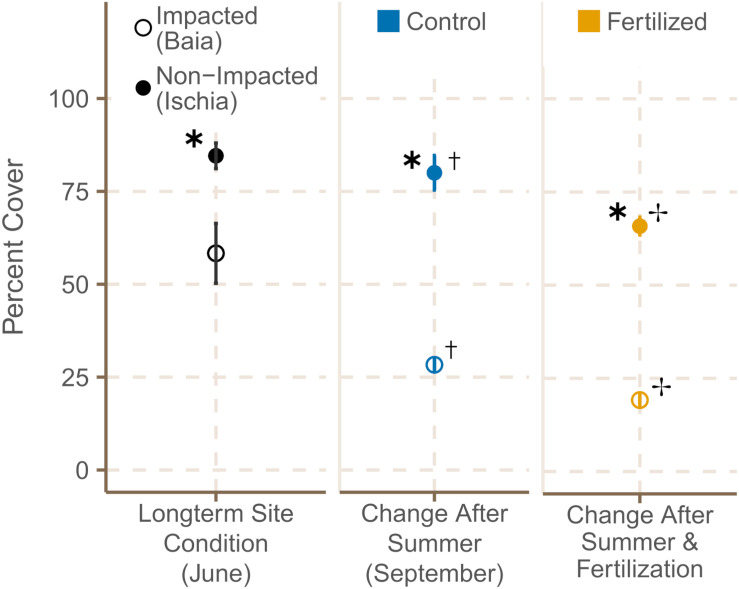
Percentage meadow cover (mean ± SE) of fertilized and control plots in June 2019 and September 2019. ✱ Shows significant differences between impacted (Baia) and non- impacted (Ischia) areas. ✝ Indicates if there seasonal differences between June and September and ✢ states if there are differences between control and fertilized plots.

Meadow cover in sites around Ischia was significantly higher with a mean of 84.6 ± 2.4% compared to sites of Baia with a mean of 58.3 ± 5.7% at the start of the experiment [Figure 2; χ^2^(1) = 8.08, *p* = 0.0045; Supplementary Table 4A]. From June to September seagrass cover decreased significantly in both locations [χ^2^(1) = 8.42, *p* = 0.0037; Supplementary Table 5], but still remained significantly higher in the two sites off Ischia compared to Baia in September [χ^2^(1) = 16.42, *p* = 0.0001; Supplementary Table 4A]. LME models additionally indicated that seagrass cover decreased even further with fertilization independent of meadow location [χ^2^(1) = 14.77, *p* = 0.0001; Supplementary Table 4A]. All significant effects reported from the LME models based on the LR tests were supported by the AICc based model comparison, meaning the model containing the tested variable (in this case condition or fertilization) was the model with the lowest AICc units and with the highest AICcWt compared to the null (or other candidate) model(s) (Supplementary Table 4A). This holds for all further analysis reported below.

**FIGURE 3:**
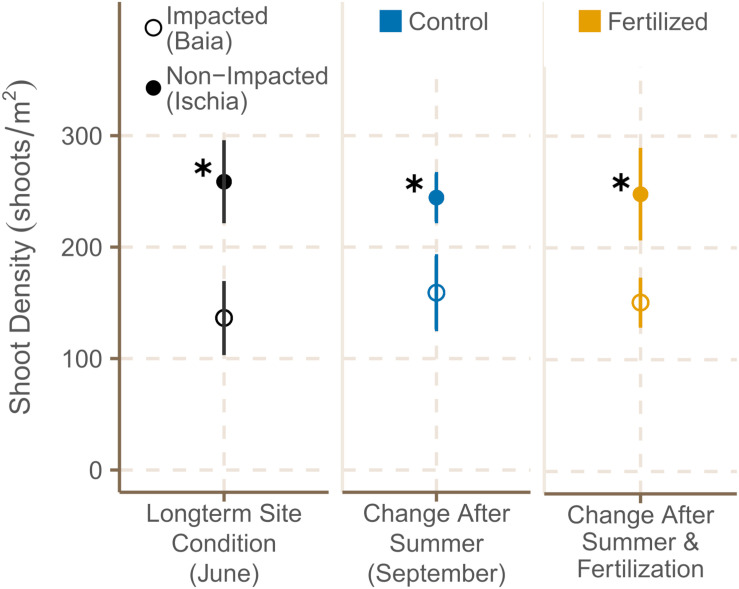
Shoot density [shoots m^2^] (mean ± SE) of fertilized and control plots in June 2019 and September 2019. ✱ Shows significant differences between impacted (Baia) and non- impacted (Ischia) areas.

Shoot density was significantly higher in meadows around Ischia than in areas already under anthropogenic pressures [Figure 3; χ^2^(1) = 10.56, *p* = 0.0012], but similar at the different sites of each location in June 2019 (see Supplementary Table 3 for data on all subsites). After the four month period, pristine sites around Ischia had still 1.6 times more shoots than impacted sites at Baia [χ^2^(1) = 6.75, *p* = 0.0094; Supplementary Table 4B]. However, fertilization had no significant effect on shoot density (Figure 3).

#### Epiphyte Cover

**FIGURE 4:**
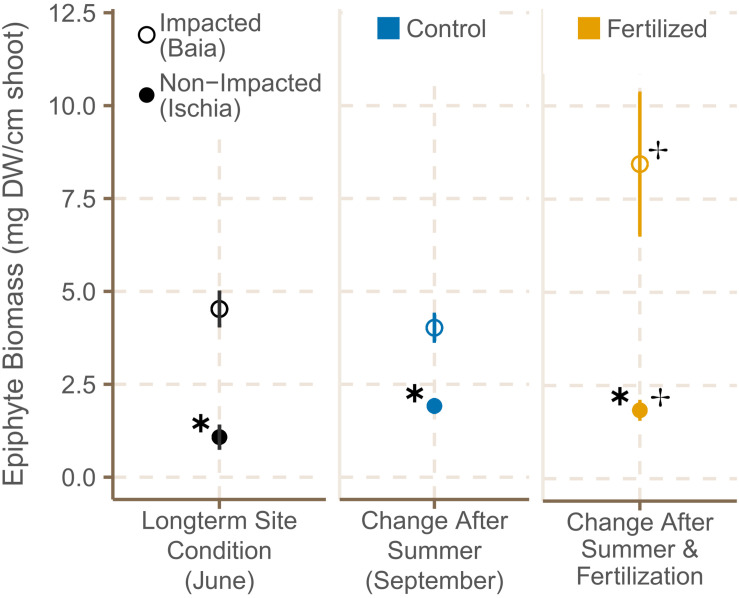
Average epiphyte biomass (±SE) of *P. oceanica* shoots in control and fertilized plots at the different sites in June and September 2019. ✱ Shows significant differences between impacted (Baia) and non- impacted (Ischia) areas and ✢ indicates if there are differences between control and fertilized plots.

Epiphyte biomass [mg (DW) epiphytes cm^–2^ shoot^–1^] in June 2019 was approximately fourfold higher in seagrass meadows in Baia (4.48 mg cm^–2^ shoot, on average) than in meadows around Ischia (1.07 mg cm^–2^ shoot, on average) [Figure 4; χ^2^(1) = 7.44, *p* = 0.0064; Supplementary Table 4C]. By September 2019, epiphyte load on leaves of plants in high impacted sites was still significantly higher than the one in low impacted sites [χ^2^(1) = 5.04, *p* = 0.0281]. In contrast to Baia, epiphyte cover remained relatively low in sites around Ischia over the course of the experiment (Figure 4). All fertilized plots had a higher epiphyte biomass [Figure 4; χ^2^(1) = 7.44, *p* = 0.0064; Supplementary Table 4C] and epiphyte biomass from plants in high impacted sites even doubled. There was also a crossover interaction showing that epiphyte cover in low impacted sites increased slightly more in control plots rather than in fertilized ones [χ^2^(1) = 7.79, *p* = 0.0053; Supplementary Table 4C).

#### Morphological Trait Response

Plants growing in low impacted meadows around Ischia had fewer leaves when compared to seagrasses growing in the high impacted sites, in June [Table 1; χ^2^(1) = 8.70, *p* = 0.0032; Supplementary Table 6A]. However, the number of leaves per shoot decreased over the course of the experiment at both locations [χ^2^(1) = 13.29, *p* = 0.0003; Supplementary Table 7], but seagrasses growing off Ischia still had more leaves per shoot by September [Table 1; χ^2^(1) = 9.62, *p* = 0.0019]. According to predictions of the best model, plants from Ischia also had taller leaves [χ^2^(1) = 10.83, *p* = 0.001; Supplementary Table 6B] and a significantly higher leaf canopy height [Table 1; χ^2^(1) = 7.55, *p* = 0.006] than seagrasses in high impacted sites, in June. Leaf length [χ^2^(1) = 69.77, *p* ≤ 0.0001] and the maximum canopy height [χ^2^(1) = 32.82, *p* ≤ 0.0001; Supplementary Table 7] showed a significant seasonal reduction from June to September in both locations (Table 1). *P. oceanica* shoots in low impacted sites still had significantly taller leaves [χ^2^(1) = 9.12, *p* = 0.0025) and a higher leaf canopy at the end of the experiment [χ^2^(1) = 11.39, *p* = 0.0001; Supplementary Table 6C] than plants growing in highly impacted sites.

**TABLE 1:** Mean (±SE) values of morphological traits from *P. oceanica* meadows (*n* = 12) from the anthropogenically impacted sites (Baia- Gulf of Pozzuoli) and low impacted sites (Ischia) in control (*n* = 6) and fertilized (*n* = 6) plots.

Morphological traits	Area	June 2019	September 2019	
			Control	Fertilized
No. leaves per shoot	Baia	5.56 ± 0.12	3.67 ± 0.18	3.61 ± 0.23
	Ischia	4.61 ± 0.15	4.72 ± 0.21	4.22 ± 0.17
Leaf height	Baia	549.71 ± 16.70	273.03 ± 27.08	274.05 ± 27.72
	Ischia	704.87 ± 22.12	435.84 ± 36.48	370.88 ± 33.73
Max. leaf canopy height	Baia	825.08 ± 17.12	490.17 ± 35.80	512.67 ± 33.84
	Ischia	961.39 ± 28.69	824.72 ± 32.15	694.44 ± 36.42
LAI	Baia	3.32 ± 0.24	1.47 ± 0.19	1.64 ± 0.17
	Ischia	7.44 ± 0.47	3.79 ± 0.36	3.36 ± 0.29
Leaf width	Baia	10.0 ± 0.0	11.03 ± 0.23	11.20 ± 0.22
	Ischia	9.11 ± 0.12	9.78 ± 0.19	9.69 ± 0.23
Leaf area	Baia	549.71 ± 60.70	301.78 ± 30.46	311.22 ± 32.09
	Ischia	646.33 ± 21.44	423.60 ± 35.59	353.70 ± 32.33
Leaf area per shoot	Baia	2394.33 ± 85.98	1017.24 ± 120.44	1080.31 ± 107.18
	Ischia	2813.03 ± 135.03	1540.36 ± 129.67	1334.43 ± 108.38

*Fertilization did not show any effects on morphological parameters except for maximum leaf canopy height, which was lower in fertilized plots in seagrasses around Ischia. Leaf height, maximum leaf canopy height, leaf width, leaf area, and leaf area per shoot are expressed in mm, while LAI is expressed as m^2^ leaf area per shoot x shoot density per m^2^.*

According to predictions of the best model, leaves of plants growing in Baia were wider than leaves from plants in Ischia [χ^2^(1) = 5.14, *p* = 0.0234; Supplementary Table 6D]. However, leaf width significantly increased over the course of the experiment independent of their location [Table 1; χ^2^(1) = 21.79, *p* ≤ 0.0001; Supplementary Table 7], but leaves were still wider in plants from Baia in September [χ^2^(1) = 5.96, *p* = 0.0146]. LME models further indicated that leaf area, leaf area per shoot as well as LAI were significantly larger in seagrasses from low impacted sites than in plants from Baia in June as well as in September 2019 (Supplementary Tables 6E,F). However, LAI [χ^2^ (1) = 23.26, *p* ≤ 0.0001] as well as leaf area [χ^2^(1) = 53.60, *p* ≤ 0.0001] and leaf area per shoot [χ^2^ (1) = 47.86, *p* ≤ 0.0001; Supplementary Table 7] did decrease over the time of the experiment independent of their location (Table 1).

Fertilization did not result in major changes in morphological traits (Table 1). Only maximum canopy height was significantly reduced in low impacted sites off Ischia as a response to the additional nutrient input [χ^2^ (1) = 4.99, *p* = 0.001; Supplementary Table 6C].

#### Carbon, Nitrogen and Phosphorous Content and Stable Isotope (δ^13^C and δ^15^N) Composition

##### Leaves

Carbon content was not significantly different in leaves of plants from the four sites at the start of the experiment (Table 2), but increased significantly over time at both locations [χ^2^ (1) = 20.05, *p* ≤ 0.0001; Supplementary Table 9]. Nitrogen content in leaves was significantly lower in plants growing around Ischia at the start [χ^2^(1) = 5.92, *p* = 0.0149] and at the end of the experiment [χ^2^(1) = 12.00, *p* = 0.0005; Supplementary Table 8C]. Consequently, C:N ratios in leaf tissues of seagrasses around Ischia were higher than those of plants in Baia at the beginning [χ^2^ (1) = 4.72, *p* = 0.0298] and at the end of the experiment [χ^2^(1) = 12.69, *p* = 0.0004; Supplementary Table 8A]. According to predictions of the best model, fertilization significantly reduced Leaf C:N ratios at both locations (Table 2 and Supplementary Table 8A).

**TABLE 2:** Mean (±SE) values of nutrient content in leaf and rhizome tissues from *P. oceanica* meadows (*n* = 12) from the anthropogenically impacted sites (Baia- Gulf of Pozzuoli) and low impacted sites (Ischia) in control (*n* = 6) and fertilized (*n* = 6) plots.

Biochemical traits	Area	June 2019	September 2019	
			Control	Fertilized
Leaf C	Baia	31.72 ± 0.30	34.90 ± 0.73	35.00 ± 0.54
	Ischia	31.87 ± 0.18	33.81 ± 0.41	34.76 ± 0.43
Rhizome C	Baia	39.75 ± 0.40	42.30 ± 1.46	39.80 ± 0.25
	Ischia	39.84 ± 0.23	41.13 ± 0.39	42.42 ± 1.53
Leaf N	Baia	1.79 ± 0.06	2.10 ± 0.25	2.20 ± 0.20
	Ischia	1.18 ± 0.08	1.02 ± 0.10	1.31 ± 0.10
Rhizome N	Baia	3.08 ± 0.29	5.10 ± 0.38	3.70 ± 0.38
	Ischia	1.64 ± 0.17	2.26 ± 0.18	3.26 ± 0.40
Leaf δ^13^C	Baia	−12.73 ± 0.20	−14.10 ± 0.48	−13.40 ± 0.29
	Ischia	−13.20 ± 0.26	−12.28 ± 0.53	−12.94 ± 0.30
Rhizome δ^13^C	Baia	−12.90 ± 0.17	−13.20 ± 0.11	−12.90 ± 0.18
	Ischia	−13.29 ± 0.14	−12.71 ± 0.22	−13.11 ± 0.12
Leaf δ^15^N	Baia	6.54 ± 0.22	5.70 ± 0.28	4.90 ± 0.23
	Ischia	6.13 ± 0.35	4.80 ± 0.52	4.17 ± 0.34
Rhizome δ^15^N	Baia	6.58 ± 0.12	6.20 ± 0.07	5.80 ± 0.22
	Ischia	6.00 ± 0.24	5.37 ± 0.14	5.25 ± 0.25
Leaf C:N ratio	Baia	14.38 ± 1.50	17.80 ± 1.87	16.70 ± 1.43
	Ischia	28.50 ± 1.98	34.42 ± 2.83	27.43 ± 2.12
Rhizome C:N ratio	Baia	17.98 ± 0.72	8.60 ± 0.86	11.50 ± 1.42
	Ischia	28.22 ± 3.95	18.77 ± 1.52	14.05 ± 1.75
Leaf P	Baia	1038.25 ± 113.28	904.80 ± 304.93	727.00 ± 67.10
	Ischia	618.08 ± 40.48	654.00 ± 69.42	722.83 ± 67.10
Rhizome P	Baia	647.00 ± 123.47	1790.70 ± 219.08	1496.30 ± 257.00
	Ischia	571.83 ± 76.87	1183.17 ± 74.93	1685.83 ± 201.48

*C and N content are expressed as percentages, while δ^13^C and δ^15^N are in parts per thousands. P content is in μg g–^1^.*

Leaf P content was approximately three times higher in plants from Baia as in plants from Ischia in June [χ^2^ (1) = 7.75, *p* = 0.0054; Supplementary Table 8D] and slightly decreased over the course of the experiment [χ^2^(1) = 0.8625, *p* = 0.03; Supplementary Table 9]. However, leaf P content did not differ significantly between locations in September probably due to the high site- specific variability (see Supplementary Table 3 for subsite variation). Plants from impacted areas had significantly reduced δ^13^C values in their leaves by September when compared to seagrasses from Ischia [χ^2^(1) = 5.70, *p* = 0.0170; Supplementary Table 10A].

##### Rhizomes

Carbon and phosphorous contents in rhizomes of plants from both locations were not significantly different and did also not change with the addition of fertilizer (Supplementary Tables 8G,H). However, nitrogen contents in rhizomes of seagrasses from Baia were significantly higher than the ones of plants off Ischia in June [χ^2^(1) = 9.73, *p* = 0.0018; Table 2] as well as in September [χ^2^(1) = 5.83, *p* = 0.00157; Supplementary Table 8F]. Seagrasses growing in low impacted sites had consequently significantly higher C:N ratios in their rhizomes compared to seagrasses from Baia at the beginning [χ^2^(1) = 7.56, *p* = 0.0059] and at the end of the experiment [χ^2^(1) = 4.80, *p* = 0.0284; Supplementary Table 8E]. Nitrogen [χ^2^(1) = 12.80, *p* = 0.0001], carbon [χ^2^(1) = 47.86, *p* ≤ 0.0001] as well as phosphorous content [χ^2^(1) = 27.63, *p* ≤ 0.0001; Supplementary Table 9] significantly increased in rhizome tissues of seagrasses from both locations over the course of the experiment. In contrast, C:N ratios in rhizomes decreased significantly from June to September in all plots independently from their location [χ^2^(1) = 6.89, *p* = 0.0087; Supplementary Table 9].

The addition of fertilizer had opposing effects on rhizome nutrient contents depending on the location of the seagrass meadows. Plants in the high impacted sites of Baia had significantly lower rhizome N contents in fertilized plots, while the %N in rhizomes of seagrasses in the low impacted sites increased with fertilization [χ^2^(1) = 12.39, *p* = 0.0004; Supplementary Table 8F]. Additionally, plants in fertilized plots in Baia had higher C:N ratios in rhizomes, while fertilization resulted in reduced C:N ratios in rhizomes of seagrasses from low impacted sites [χ^2^(1) = 9.51, *p* = 0.0020; Supplementary Table 8E].

There were no differences in δ^15^N and δ^13^C isotope values neither in leaf nor in rhizome tissues of seagrasses from Ischia and Baia in June 2019. Fertilization only resulted in lower δ^15^N content in leaf tissues of seagrasses from both locations, with a more pronounced reduction in plants from Ischia (Supplementary Table 10B). Moreover, rhizome δ^15^N values declined significantly over the course of the experiment [χ^2^(1) = 5.63, *p* = 0.0177; Supplementary Table 11], resulting in significantly lower δ^15^N values in rhizomes of plants from Ischia by the end of the experiment [χ^2^(1) = 6.59, *p* = 0.0103].

#### Non-structural Carbohydrates

##### Leaves

**FIGURE 5:**
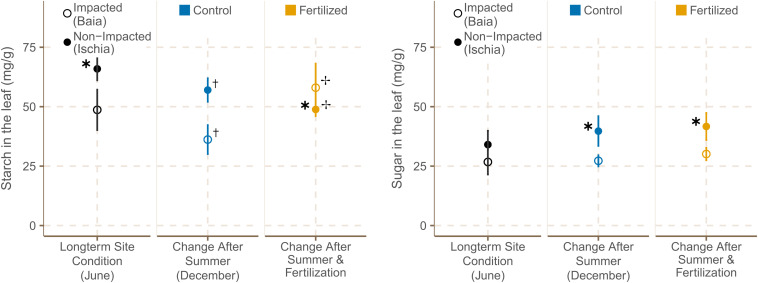
Starch and sugar concentrations [mg g^− 1^] in leaves (mean ± SE) of *P. oceanica* plants (*n* = 6) in fertilized and control plots from different sites in June and September 2019. ✱ Shows significant differences between impacted (Baia) and non- impacted (Ischia) areas. ✝ Indicates if there seasonal differences between June and September and ✢ states if there are differences between control and fertilized plots.

Starch concentration in the leaves of *P. oceanica* plants were significantly higher in low impacted sites at the start of the experiment [χ^2^(1) = 5.97, *p* = 0.0146; Supplementary Table 12B]. From June to September, starch concentration in leaf tissue was significantly reduced in all sites independently from their location [Figure 5; χ^2^(1) = 6.22, *p* = 0.0126; Supplementary Table 13]. The addition of fertilizer reduced the starch contents in leaves from seagrasses in Ischia even further, whereas plants from Baia had significantly increased starch concentrations when nutrients were added [χ^2^(1) = 6.46, *p* = 0.0110; Supplementary Table 12B].

In contrast, according to predictions of the best model, leaf sugar concentration showed a slight trend of increasing over the course of the experiment in all sites (Supplementary Table 13) and leaves of seagrasses from low impacted sites around Ischia had significantly higher leaf sugar concentration than plants from Baia by the end of the experiment [Figure 5; χ^2^(1) = 4.53, *p* = 0.0333; Supplementary Table 12A].

##### Rhizomes

**FIGURE 6:**
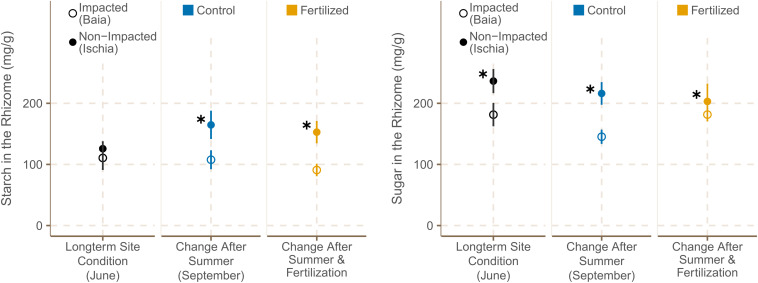
Starch and sugar concentrations [mg g^– 1^] in rhizomes (mean ± SE) of *P. oceanica* plants (*n* = 6) in fertilized and control plots from different sites in June and September 2019. ✱ Shows significant differences between impacted (Baia) and non- impacted (Ischia) areas.

Starch concentrations in the rhizomes of *P. oceanica* meadows did not differ significantly among the sites in Baia (Gulf of Pozzuoli) and sites around Ischia at the start of the experiment (Figure 6). However, by September, the rhizome starch concentration of *P. oceanica* in the high impacted sites was significantly lower than those of the Ischia plants [χ^2^ (1) = 7.49, *p* = 0.0062; Supplementary Table 12D].

The average concentration of soluble sugars was significantly higher in rhizomes in the plants from Ischia compared to the ones of Baia in June 2019 [Figure 6; χ^2^(1) = 7.46, *p* = 0.0063], and according to predictions of the best model showed a trend of declining over the time of the experiment in all plots at all sites (Supplementary Table 13). Rhizome sugar content was still significantly higher in seagrasses from low impacted sites by September 2019 [χ^2^(1) = 5.63, *p* = 0.0062; Supplementary Table 12C].

The short- term fertilization had no statistically significant effect on starch or sugar content even though there was a different trend in the response to fertilization between the high impacted and low impacted sites as shown by the low deviance explained the models (Supplementary Table 12). Seagrass rhizomes of high impacted sites showed a slight decrease in rhizome starch concentration with fertilization, while plants in low impacted sites showed a trend of increasing their rhizome starch concentration with fertilization (Figure 6).

### Comparison of Relative Changes With Season and Fertilization Across Population and Individual Plant Traits

To better compare and visualize differences across all population level metrics and individual plant morphological and biochemical traits due to season and to fertilization effects, we calculated the relative percent differences of all responses in September compared to in June (Table 3). Morphological traits were mainly impacted by season than by the additional fertilization. All morphological parameters got significantly reduced over time with more pronounced negative responses in plants from Baia. Population metrics of seagrasses generally displayed negative trends as well, except for epiphyte load which mainly increased over time and with fertilization. Biochemical traits showed a mix of either negative or positive trends, depending on the interaction of fertilization and on the level of impact of the sites.

**TABLE 3:** Relative percent change (mean ± SE) over the season (shown under Control) and relative percent difference (mean ± SE) with the interactive effects of season and fertilization (as shown under Fertilized) in the measured parameters in rhizome and leaf tissues of seagrass plants in control (*n* = 6) and fertilized (*n* = 6) plots from the anthropogenically impacted sites (Baia, Gulf of Pozzuoli) and low impacted sites (Ischia) in June compared to control (*n* = 6) and fertilized (*n* = 6) plots in September.

Traits	Site	Changes until September 2019 (%)	
		Control	Fertilized
**Population level**			
Cover	Baia	**−54.05 ± 2.70**	**−65.15 ± 2.80**
	Ischia	**−2.04 ± 5.70**	**−24.76 ± 2.72**
Shoot density	Baia	+29.71 ± 15.48	+0.60 ± 8.00
	Ischia	−6.24 ± 8.23	−4.12 ± 15.43
Epiphytes	Baia	−11.03 ± 8.77	**+86.16 ± 41.86**
	Ischia	+55.65 ± 13.25	**+82.23 ± 27.71**
**Morphological**			
No. leaves per shoot	Baia	**−32.65 ± 3.32**	**−**36.28 ± 4.07
	Ischia	**+1.62 ± 7.86**	**−**7.32 ± 6.56
Leaf height	Baia	**−6.18 ± 4.66**	+0.16 ± 4.84
	Ischia	**+1.15 ± 4.56**	**−**1.75 ± 4.11
Max. leaf canopy height	Baia	**−38.92 ± 4.46**	**−**39.52 ± 3.99
	Ischia	**−14.93 ± 5.75**	**−26.36 ± 6.69**
LAI	Baia	**−49.73 ± 6.39**	**−**55.75 ± 4.69
	Ischia	** −47.76 ± 8.59**	**−**55.05 ± 6.73
Leaf width	Baia	**+10.28 ± 2.30**	+ 12.00 ± 2.16
	Ischia	**+7.94 ± 3.64**	+6.40 ± 4.29
Leaf area	Baia	**−6.85 ± 4.54**	+0.16 ± 4.84
	Ischia	**+1.27 ± 4.86**	**−**1.78 ± 4.32
Leaf area per shoot	Baia	**−56.45 ± 5.16**	**−**55.95 ± 4.37
	Ischia	**−42.82 ± 8.34**	**−**53.48 ± 6.54
**Biochemical**			
Leaf C	Baia	**+11.51 ± 2.33**	+9.05 ± 1.67
	Ischia	**+6.05 ± 1.27**	+9.13 ± 1.34
Rhizome C	Baia	**+7.14 ± 3.70**	**−**0.72 ± 0.63
	Ischia	**+3.14 ± 0.97**	+6.55 ± 3.84
Leaf N	Baia	+15.71 ± 13.80	+23.09 ± 11.21
	Ischia	−9.05 ± 9.15	+5.41 ± 8.19
Rhizome N	Baia	**+70.80 ± 12.82**	**+15.37 ± 11.92**
	Ischia	**+65.66 ± 13.10**	**+71.01 ± 21.21**
Leaf δ^13^C	Baia	+10.10 ± 3.75	+5.98 ± 2.31
	Ischia	**−**5.35 ± 4.08	**−3.49 ± 2.24**
Rhizome δ^13^C	Baia	**−**0.38 ± 0.85	+0.27 ± 1.44
	Ischia	**−**4.66 ± 1.68	**−1.11 ± 0.91**
Leaf δ^15^N	Baia	−9.60 ± 4.34	**−27.23 ± 3.46**
	Ischia	−12.45 ± 9.50	**−38.48 ± 5.08**
Rhizome δ^15^N	Baia	**−4.10 ± 1.07**	**−**12.67 ± 3.24
	Ischia	**−8.95 ± 2.31**	−14.15 ± 4.07
Leaf C:N ratio	Baia	+1.04 ± 10.65	**−9.32 ± 7.77**
	Ischia	+12.73 ± 9.26	**+3.92 ± 8.03**
Rhizome C:N ratio	Baia	**−44.33 ± 5.53**	**−13.23 ± 10.71**
	Ischia	**−45.50 ± 4.42**	**−36.07 ± 7.94**
Leaf P	Baia	**−11.64 ± 29.78**	**−**30.93 ± 19.48
	Ischia	**+10.54 ± 11.73**	+12.15 ± 10.41
Rhizome P	Baia	**+310.39 ± 50.21**	+74.47 ± 29.96
	Ischia	**+130.64 ± 14.61**	+167.31 ± 31.95
Leaf sucrose	Baia	+13.53 ± 9.75	−1.97 ± 8.87
	Ischia	+31.81 ± 20.74	+9.77 ± 14.98
Leaf starch	Baia	**−40.17 ± 9.81**	**+57.07 ± 27.03**
	Ischia	**−13.22 ± 7.59**	**−26.09 ± 4.30**
Rhizome starch	Baia	+11.07 ± 14.38	**−**26.98 ± 7.14
	Ischia	+25.43 ± 16.53	+26.60 ± 13.99
Rhizome sucrose	Baia	**−**20.89 ± 5.58	+1.29 ± 5.52
	Ischia	**−**12.69 ± 6.91	**−**9.83 ± 12.33

*Statistically significant values are in bold.*

## Discussion

Our results show a significant impact of local anthropogenic nutrient pressure on *Posidonia oceanica* meadows in the mid- western Mediterranean. With a larger number of spatial or temporal replicates, it is likely that the effects of fertilization would be reflected in more significant changes in seagrass indicators, but due to both time and logistic constraints further replication was not possible in this study. Despite these limitations, our findings identify useful indicators of nutrient and temperature stress for meadows of *Posidonia oceanica*, which provides us with a good example of a typical response of a large-bodied, slow growing seagrass undergoing various levels of local anthropogenic stressors (see Table 4 for simplified summary of indicators). In contrast to the small-bodied, fast growing species *Halophila stipulacea*, from Part II of our study, which seemed to be able to adapt to eutrophic conditions (Helber et al., unpublished), *Posidonia oceanica* from the impacted sites showed negative responses while enduring unusually high summer temperatures that were exacerbated even more by the additional nutrient enrichment provided in our study. This was shown by the decline in percentage cover, leaf area index (LAI), rhizome starch and sugar concentrations and the drastic increases in epiphyte biomass. Short-term effects of high temperature exposure during the summer months were also found in the less impacted sites, but with mixed effects of nutrient enrichment.

**TABLE 4:**
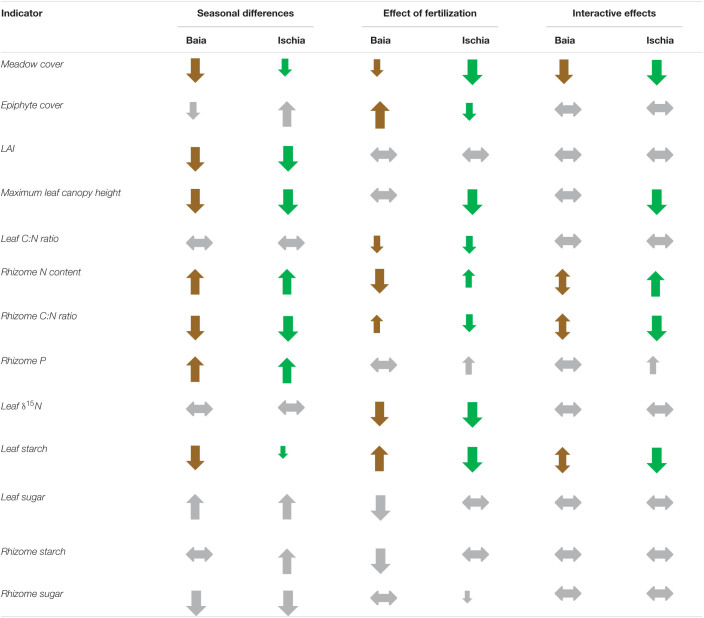
Significant differences in the most important investigated parameters caused by time (season and/or temperature) and by the addition of fertilizer as well as their interactive effects.

*Statistically significant effects on seagrasses from the high impacted sites in Baia are depicted in brown color, while statistically significant effects on plants from the low impacted sites around Ischia are represented in green colors. The direction and size of the arrows represent the direction and strength of the effects (according to Table 3), respectively. The gray arrows represent no significant effects in either direction.*

The most responsive indicators we detected for nutrient stress in this seagrass can be used to monitor further impacts of nutrient inputs to these seagrass meadows and provide management options to minimize future human interference on these important ecosystems.

### Locally Driven Environmental Impacts Across Sites

#### Nutrient Concentrations and Water Quality

Nutrient concentrations in the water column and in the porewater were found to give inconclusive results, especially since sampling was only done at two time points. This was reported in previous studies: rapid dilution processes as well as fast transfer through the food web to higher trophic levels through phytoplankton grazing can make it difficult to detect signs of eutrophication via dissolved nutrients (Dalsgaard and Krause-Jensen, 2006; Holmer et al., 2008; Pitta et al., 2009). In addition, nutrients are often rapidly taken up by epiphytes of *P. oceanica* leaves as well as macroalgae in the seagrass meadow (Ruiz et al., 2010; Apostolaki et al., 2011). Micro- and macroalgae bioassays were proposed by Dalsgaard and Krause-Jensen (2006) as a reliable detection method for higher nutrient loads caused by fish farming activities. Similarly, in our study, epiphyte cover may be used as such a proxy (but see discussion below).

#### Epiphyte Cover as an Indicator of Short- and Long-Term Nutrient Enrichment and Thermal Stress

The epiphyte community of sites in Baia was characterized by large development of brown algae as reported for sites subjected to human pressure, whereas the sites off Ischia showed a mature epiphyte community consisting of red algae (Lepoint et al., 2007; Balata et al., 2008; Giovannetti et al., 2010). Nutrient enrichment and exposure to sewage was shown to drastically change the species composition of epiphytes (Balata et al., 2008; Prado et al., 2008; Giovannetti et al., 2010). In addition, previous studies (Frankovich and Fourqurean, 1997; Ferdie and Fourqurean, 2004; Kružić, 2008; Balata et al., 2010) confirmed that increased dissolved nutrient concentrations can promote epiphyte overgrowth on seagrass leaves. However, the reliability of epiphyte load as an indicator for eutrophication depends strongly on the interaction with herbivory on epiphytes, as increased grazing pressure can control the abundance of epiphytes (Ruiz et al., 2001; Heck et al., 2006). Herbivores have been observed to prefer adult leaves with a higher epiphyte load and higher nitrogen content (Peirano et al., 2001). Although we did not directly quantify herbivory on epiphytes in our study, it did not seem to have much effect on epiphyte biomass which was significantly higher in sites already subjected to anthropogenic stress in Baia and also differed between fertilized and control plots. The continuous nutrient input received by the seagrass meadows in Baia over a longer time scale possibly outweighs herbivory and as a result shifted the control of epiphytes from top–down to bottom–up.

Temperature and light availability are assumed to be the main drivers that facilitate the summer increase in epiphyte load (Prado et al., 2008; Apostolaki et al., 2011; Peirano et al., 2011). Here, we observed a slightly higher increase in epiphyte load from June to September as recorded in previous studies (Prado et al., 2008; Apostolaki et al., 2011; Peirano et al., 2011), but the epiphyte biomass of seagrass leaves in Baia was almost five-fold higher in fertilized plots in September 2019 than the one found in plants off Ischia. While a modest epiphyte cover protects the leaves from damage due to ultraviolet radiation (Trocine et al., 1981; Nelson and Waaland, 1997), an increase in epiphyte biomass has several negative impacts on the seagrass plant. Epiphytes reduce light reaching the leaf surface and block leaf nutrient uptake outcompeting seagrass plants for nutrients (Ruiz et al., 2001; Cornelisen and Thomas, 2004; Apostolaki et al., 2012). In a related study, the photo-chemical performance in *P. oceanica* was shown to decrease in seagrasses from both low and high impacted locations during nutrient enrichment in mesocosm experiments (Pazzaglia et al., 2020); thus reduced light availability possibly due to epiphyte growth could have been one of the reasons. This can impact seagrass growth rates and negatively influence the carbon budget of plants exposed to eutrophication (Ruiz et al., 2001; Cornelisen and Thomas, 2004; Apostolaki et al., 2012).

Overall, our study confirms that epiphyte biomass in *P. oceanica* is more sensitive to changes in nutrient availability compared to the host plants or the whole community and is therefore an ideal indicator to identify seagrass meadows in which plant stress is likely to occur.

### *P. oceanica* Population Response to Nutrient and Thermal Stress

The percentage cover of *P. oceanica* meadows already experiencing high anthropogenic pressure dropped drastically from June to September 2019 regardless of further nutrient enrichment in comparison to less impacted sites which showed only minor changes. Similar low shoot densities as the one in the Gulf of Pozzuoli have been observed for meadows in the vicinity of fish farms (Delgado et al., 1999; Ruiz et al., 2010). The lower shoot density recorded in a few plots within the low impacted sites, could possibly be the result of anchor damage (S. Helber, personal observation). Percent cover was also affected in all fertilized plots compared to non-fertilized plots within each site, particularly at the impacted location. Sewage input as well as high summer temperatures, have been identified in other studies for a decrease in shoot density (Delgado et al., 1999; Mayot et al., 2005; Marbà et al., 2014).

Shoot density was significantly higher in the low impacted sites of Ischia compared to the high impacted sites in Baia during both sampling periods. No significant change in shoot density over the experimental period could been detected, likely due to high variability in shoot counts as observed in other studies (Kletou, 2019; Zulfikar and Boer, 2020). This is not surprising, since shoot density in meadows at 10 m depth does not show strong seasonality (Peirano et al., 2011), and can remain fairly constant over long time periods (Kletou, 2019). In the present study, the lower response in shoot density to additional fertilization compared to percent cover shows that this measure is less reliable as an indicator for detecting nutrient stress in *P. oceanica*.

*Posidonia oceanica* meadows often contain two types of shoots, the most common ones being vertical shoots and the less frequent ones being horizontal or apical shoots (Hemminga and Duarte, 2000). Interestingly, we could barely find any vertical shoots in September 2019 at Site 1 in the plots in Baia. This might further indicate that the plants are stressed and the condition of the meadow will deteriorate further. Plants try to spread horizontally to recover and find more favorable conditions (Meinesz and Lefevre, 1984). Indeed, stressed meadows will prioritize apical shoots survival in order to secure population persistence and spread to more favorable habitats (Ruocco et al., 2020). In order to respond to temperature stress, plants can perform escape strategies other than favoring the growth of apical shoots, and can invest resources in flowering, seeking for spreading through sexual reproduction (Ruiz et al., 2018; Marín-Guirao et al., 2019). Nevertheless, flowering was not observed during the experimental period.

Rhizomes of plants from Baia were more brittle, easy to break and had a distinct hydrogen sulfide smell, whereas rhizomes from seagrasses around Ischia were firm and hard to break off (S. Helber, personal observation). Shoot mortality in *P. oceanica* as well as impacts on the photosynthetic rates of adult *P. oceanica* plants and their seedlings have been shown to increase during periods of high temperature in the summer months, in particular when temperatures reach 27°C for several days or weeks (Mayot et al., 2005; Díaz-Almela et al., 2009; Marbà and Duarte, 2010; Guerrero-Meseguer et al., 2017). Our data show that seagrass plants were exposed to temperatures over 27°C for 1–3 weeks at our experimental sites. Moreover, maximum temperatures recorded at 10 m depth were as high as 28.26°C, similarly to temperatures previously recorded for the extremely hot summer in 2003 (Garrabou et al., 2009).

Overall, our results confirm other studies which found increases in shoot mortalities of *P. oceanica* plants when seawater temperatures exceeded 27°C (Marbà and Duarte, 2010). Other studies showed that a one month exposure of *P. oceanica* seedlings above 29°C led to a seedling mortality rate of 33% and to a leaf mortality rate of 60% (Guerrero-Meseguer et al., 2017). Previous heat waves in the Mediterranean caused already high shoot mortalities from which plants in deeper areas were not able to recover within years (Marbà and Duarte, 2010). Future heat waves and warming are expected to reach even higher temperatures thereby posing a significant threat to *P. oceanica* populations (IPCC., 2007, 2014; Giorgi and Lionello, 2008). Our study further highlights that the overall magnitude of thermal stress effects on the population level can be exacerbated by the degree of anthropogenic nutrient supply, as those sites with higher impact were more significantly affected by the summer heat wave compared to low impacted sites.

### Individual Plant Responses as Stress Indicators

#### Elemental (C, N, and P) and Stable Isotope (δ^13^C and δ^15^N) Tissue Content

Tissue content of nitrogen, carbon and phosphorous are valid descriptors of the nutritional status of seagrasses. The nitrogen content in leaves and rhizomes of seagrasses provides information about their long-term nutrient exposure and has been found to be higher in *P. oceanica* tissues at sites that receive higher nutrient inputs (Udy and Dennison, 1997; Invers et al., 2004; Fourqurean et al., 2006). In our study, an increase in %N content was first visible in the rhizomes of plants in fertilized plots growing in low impacted areas, highlighting the role of the rhizomes as a main location for N storage in this species (Invers et al., 2004; Fourqurean et al., 2006). Moreover, %P content increased significantly in the rhizomes of the seagrasses at both sites from June to September and the increase was even more pronounced in fertilized plots of meadows around Ischia suggesting that seagrasses might have been P limited.

Changes in C:N ratios of plants from both locations were caused by differential uptake of nitrogen as carbon content in rhizome and leaves of seagrasses at both locations remained constant. The same results have been observed by Pazzaglia et al. (2020) indicating that seagrass plants from pristine sites and sites receiving high nutrient inputs in the Gulf of Pozzuoli (Baia) developed different nutrient-balance strategies. Additional fertilization showed no effect in nitrogen content of seagrass tissues in plants growing under eutrophic conditions, possibly because nutrient imbalances might already exist in those plants, which may respond by downregulating their nitrogen uptake (Burkholder et al., 1992; Udy and Dennison, 1997; Ruocco et al., 2018; Pazzaglia et al., 2020). *P. oceanica* from the high impacted sites seemed to have already taken up and stored inorganic forms of N following their persistent nutrient exposure as indicated by their higher N content in leaf and rhizome tissues. Uptake and assimilation of nitrate or ammonium are energetically costly and require energy for nitrate reduction as well as C skeletons to produce amino acids (Van Katwijk et al., 1997; Touchette and Burkholder, 2000). Thus, C skeletons needed to produce carbohydrate reserves are diverted for the amino acid production (Invers et al., 2004; Pazzaglia et al., 2020). In the present study, we measured lower carbohydrate reserves in *P. oceanica* rhizomes from Baia and a trend towards a further decrease of carbohydrate reserves in fertilized plots, confirming this notion.

Conversely, plants from Ischia showed a decline in their leaf starch content in the fertilized treatments, indicating a mobilization of their carbon reserves from leaves to cope with the nutrient addition, confirming what has been previously reported in a mesocosm experiment (Pazzaglia et al., 2020). Plants in fertilized plots off Ischia showed an increase of N and P content in leaf and rhizome tissues, suggesting N and P limitation for *P. oceanica* at these sites. Thus, in response to the supply of the fertilizer, these plants showed an opportunistic nutrient strategy, assimilating N and P as soon as it became available (Marín-Guirao et al., 2018; Ruocco et al., 2018).

δ^13^C and δ^15^N values obtained in this study are in the same order of magnitude as those reported in previous studies for the same species (Vizzini et al., 2003; Lepoint et al., 2004; Fourqurean et al., 2007; Mateo et al., 2010), and ranged from –10.76 to –15.48 ‰ for δ^13^C and from 2.24 to 8.12 ‰ for δ^15^N. However, δ^15^N values were at the upper limit or slightly above those previously reported, suggesting that meadows both in Baia and in Ischia may be influenced by untreated or not properly treated sewage outflows, which are known to be enriched in δ^15^N (Tomicic et al., 2001; Lepoint et al., 2004; Fernandes et al., 2009). At the same time, seagrass leaves of plants from fertilized plots showed a decrease in their δ^15^N values, confirming that the plant incorporated the additional nutrients provided by the artificial fertilizer, which have δ^15^N signatures close to 0‰ (Fourqurean et al., 2005).

#### Morphological Traits

Plants from the low impacted sites of Ischia had a higher LAI than seagrasses in Baia. This difference was even more pronounced after the summer peak in temperature. Thus, plants in Ischia possess a higher photosynthetically active area, being able to produce more substrates for growth, carbohydrate, and storage. Overall, seagrasses growing in Baia had smaller leaves and a lower leaf canopy. A reduction in leaf length has been previously observed in nutrient enrichment experiments with *P. oceanica* and *Z. marina* (Short et al., 1995; Leoni et al., 2006) as well as for plants growing in natural environments exposed to urban effluents and fish farms (Delgado et al., 1999; Ruiz et al., 2001; Balestri et al., 2004). Changes in sediment biogeochemistry (e.g., anoxia or sulphidic conditions; Delgado et al., 1999), imbalances in the internal nutrient budget or ammonium toxicity (Burkholder et al., 1992; Van Katwijk et al., 1997; Invers et al., 2004) might be responsible for the smaller leaf size in the impacted sites. Leaf canopy height was reduced in fertilized plots in meadows of Ischia, which would support the hypothesis that reduced leaf length was the result of internal nutrient imbalances. Additionally, reduced leaf length could be caused by an enhanced overgrowth of epiphytes. Leaf apexes might become more fragile and as a result break off more easily (Harlin, 1980).

Reduction in leaf growth was shown to be related to an epiphyte-induced decrease of available light (Short et al., 1995; Moore and Wetzel, 2000; Leoni et al., 2006). To compensate for the increased epiphyte overgrowth and the associated declining photosynthetic rates, seagrasses increased their number of leaves as an adaptive response to maximize their photosynthetic leaf surface (Dalla Via et al., 1998; Balestri et al., 2004; Leoni et al., 2006). In our study, plants in the impacted sites indeed had higher number of leaves per shoot than plants growing in meadows around Ischia. However, after the summer peak in temperature, the number of leaves per shoot declined in plants of Baia. The extremely high summer temperatures could have been responsible for the reduction in leaf numbers per shoot. Similarly, in *P. oceanica* leaf numbers per shoot were observed to experience a 20% reduction immediately after a disturbance occurred (Guidetti, 2001).

Morphological descriptors could be used to reveal short-term stress in seagrass meadows, but their limits of use have to be considered in terms of sampling times and reproducibility. The most valid morphological indicators for stress in our study were maximum leaf canopy height and LAI, a combined measure of leaf area, number of leaves, and shoot density, all of which had been found to decrease in other studies investigating anthropogenic impacts on seagrasses (Guidetti, 2001; Leoni et al., 2006; Lopez y Royo et al., 2011; Kletou, 2019; Zulfikar and Boer, 2020).

By combining these individual plant traits with population level metrics, such as shoot density, we might be able to make predictions about changes in primary production on the ecosystem level as these functional traits have been linked to this important ecosystem function in aquatic and terrestrial plant systems (Gustafsson and Norkko, 2018; Karamfilov et al., 2019; Zulfikar and Boer, 2020). In terrestrial ecosystems, plant height, LAI and leaf coverage had all direct positive effects on ecosystem primary production (Asner et al., 2003; Gustafsson and Norkko, 2018). LAI was often found to be the main determinant of forest gross or net primary production and is considered one of the most significant parameters in most Terrestrial Biosphere Models (Asner et al., 2003; Barr et al., 2004; Saigusa et al., 2005; Wang et al., 2019). Thus, negative changes in seagrass canopy height, LAI and shoot density might in turn have long-lasting and devastating effects on important ecosystem functions, including the carbon sequestration capacity of the seagrass system.

#### Carbohydrates

Rhizome starch content of *P. oceanica* plants around Ischia was significantly higher than the one from plants in Baia and increased from June to September, whereas the rhizomes of plants in Baia did not show any seasonality. The seasonality in carbohydrate concentration found for plants in Ischia might be crucial for survival during winter, as *P. oceanica* has a distinctive asynchrony between its carbon use (respiration and growth) and its carbon fixation through photosynthesis (Alcoverro et al., 2001). The plant accumulates carbohydrate reserves in their rhizomes during summer, which are used to survive the winter season and to support growth in early spring when solar radiation is low (Alcoverro et al., 2001). In Spring, seagrass plants take advantage of their stored starch reserves and of the high water nutrient concentrations to meet their respiratory demands and to support leaf growth (Alcoverro et al., 2001). This permits them to build a large photosynthetic biomass until summer, when irradiances are high but nutrient concentrations in the water column (Romero, 1985) as well as in the porewater are at their lowest (Alcoverro et al., 1995; 1997).

The summer months of 2019 were particularly hot with maximum temperatures reaching 28.26°C at 6.6–7.9 m water depth in meadows in the Gulf of Pozzuoli (Baia) and at 7.4–12.0 m depth 27.96°C in meadows around Ischia. These hot temperatures could have led to the lower starch concentrations in the rhizomes of the already impacted seagrass meadows while the plants in less impacted areas seemed to be able to take advantage of the additional nutrients provided by the fertilization and use it to store even more starch compounds in their rhizomes. While starch content in leaves decreased in fertilized plots of low impacted sites, it increased in fertilized plots in high impacted sites. This suggests that the strategy of starch allocation and the stress coping mechanisms of the seagrasses differ with fertilization in high impacted sites compared to low impacted sites. Plants from high impacted sites seem to store energy as starch in their leaves for the direct use of new leaf growth whereas plants from low impacted sites use the additional fertilization to store higher amounts of starch in their rhizomes.

Seagrass plants require energy in the form of carbon skeletons (e.g., sugars and starch) for the assimilation of nitrogen, which can lead to a decrease in their carbohydrate reserves of up to 50% under conditions of high nitrogen availability (Burkholder et al., 1992; Van Katwijk et al., 1997; Invers et al., 2004). This decrease has been observed to be most pronounced during autumn and winter (Invers et al., 2004) for which we have no data. However, from June to September there was a tendency towards a more pronounced decline in starch content for the fertilized plots in Baia, suggesting that future nutrient input might further threaten the persistence of these meadows. High starch concentrations in the rhizome of *P. oceanica* do not only ensure winter survival, but also increase their resilience in case of disturbances since these resources can be readily mobilized in the event of extreme climatic or grazing events (Peterson et al., 2002; Fraser et al., 2014; Nowicki et al., 2017).

The lower starch concentrations in rhizomes of plants at the impacted sites could compromise seagrass health. While seagrasses from Ischia increased their starch concentrations from June to September, there was no difference in starch concentrations from plants in Baia. Thus, they might not be able to mobilize any reserves for overwintering and regrowth when their internal carbon budget is depleted. Therefore, starch content in rhizomes is considered to be a useful indicator of health status and survival probability of seagrass plants (González-Correa et al., 2008).

During heat waves, respiration of seagrasses increases which in turn might reduce the storage of carbohydrate reserves and lead to higher mortality rates, when under high temperature stress, *P. oceanica* plants, in fact, mobilize their starch reserve from rhizomes to leaves, to cope with higher energetic demand (Marín-Guirao et al., 2018). Therefore, heat stress might have a more detrimental impact on carbohydrate reserves than nutrient availability, as indicated by our study that could not detect significant effects of fertilization on carbohydrate concentrations in the rhizomes. Higher shoot mortalities have been reported in *P. oceanica* meadows in the Mediterranean in the winter months after the heat wave in the summer 2003 and could have been the consequence of depleted carbohydrate reserves (Díaz-Almela et al., 2009). In addition, it has been demonstrated that low irradiance resulted in a decline of carbohydrate concentrations in the rhizome and also in a drop in shoot density (Romero et al., 1996; Alcoverro et al., 2001). Thus, it will properly indicate when the carbon balance of the seagrass plants starts to be compromised through heat stress and/or nutrient pollution and is thus a particularly suitable indicator of the plants metabolic status.

### Ecological Implications

*Posidonia oceanica* meadows might tolerate further heat waves in the Mediterranean, but are likely to experience substantial cutbacks in their photosynthetic performance as well as in their carbohydrate allocation for storage and growth. This weakens the plant ability to compete with other macrophytes, that are not only able to survive, but also to photosynthesize and grow within a wider range of temperatures (Klein and Verlaque, 2008; Boudouresque et al., 2009; Rotini et al., 2017; Marín-Guirao et al., 2018; Beca-Carretero et al., 2020). Additionally, further increases in ambient nutrient concentrations as well as the rising input of ammonium due to the growing demand for agriculture and mariculture off the Mediterranean coasts (Karakassis and Hatziyanni, 2000; Pusceddu et al., 2007), pose a threat to the growth and survival of *P. oceanica* meadows. Persistent genera such as *Posidonia*, *Amphibolis*, and *Thalassia* spp. are particularly vulnerable to coastal eutrophication showing often a lack of recovery following nutrient reduction (Burkholder et al., 2007; Govers et al., 2014b; Fernandes et al., 2019). On top of this, the recovery of seagrass meadows that are already exposed to local anthropogenic stressors, might be further impeded by ocean warming as evidenced by the current and previous studies (Moreno-Marín et al., 2018; Ontoria et al., 2019). Our study confirms that overgrowth of epiphytes can be a sensitive indicator of a change in ecological water quality over large spatial scales, better than any *P. oceanica* structural indicator as it shows a faster response (Delgado et al., 1999; Giovannetti et al., 2010; Kletou, 2019). Epiphyte cover on leaves is also an easy to implement and low cost indicator for monitoring programs that was already proposed as an early warning indicator for deteriorating water quality as its response is independent of climate zone (Giovannetti et al., 2010; Marbà et al., 2013; Nelson, 2017). We further identify plant indicators that monitoring efforts should focus on, and especially recommend LAI and carbohydrate concentrations (starch and sugars) in the rhizomes as indicators of stress in this seagrass species.

It is of most importance to predict and anticipate stress in this sensitive key foundation species as *P. oceanica* has very limited colonizing abilities (Meinesz and Lefevre, 1984) and is highly vulnerable due to its extremely slow growth rates and low reproductive capacity (Kendrick et al., 2005; Holon et al., 2015; Noè et al., 2020). Therefore, the recovery of *P. oceanica* in denuded areas might take decades to centuries if even possible and conservation strategies should be given the utmost priority.

## Data Availability Statement

The original contributions presented in the study are included in the article/Supplementary Material, further inquiries can be directed to the corresponding author/s.

## Author Contributions

SH, GP, HR, and MT conceived and designed the experiments. EB and MS contributed in designing the experiments. UC, AS-S, and SB assisted with the fieldwork and during sampling campaigns. SH performed the field experiment, developed the methodologies, and performed all the other laboratory analyses together with SB. EB did the statistical analysis. All the authors wrote and reviewed the manuscript.

## Conflict of Interest

The authors declare that the research was conducted in the absence of any commercial or financial relationships that could be construed as a potential conflict of interest.
