# Supplementary material for: Unusually Warm Summer Temperatures Exacerbate Population and Plant Level Response of Posidonia oceanica to Anthropogenic Nutrient Stress
Source: Front Plant Sci. 2021 Jul 5;12:662682. doi: 10.3389/fpls.2021.662682 (PMC8287906; doi:10.3389/fpls.2021.662682)
Supplement: Supplementary file 1 [file Data_Sheet_1.docx]

**Figure S1.** The left image shows the seagrass meadow in the Marine Protected Area “Regno di Nettuno” off the island of Ischia, while the right image shows seagrasses growing in discontinuous meadow patches in the high impacted site in Baia (Gulf of Pozzuoli).

**Figure S2.** Daily average temperatures of all four sites at approximately 10m water depth recorded from 11.06 to 26.09.2020. The dashed line is drawn at 27 °C as negative impacts on the photosynthetic rates of adult *P. oceanica* plants and their seedlings were observed when seawater temperatures reach 27 °C and beyond (Marbà and Duarte 2010; Guerrero-Meseguer, et al., 2017).
