# Supplementary material for: Unusually Warm Summer Temperatures Exacerbate Population and Plant Level Response of Posidonia oceanica to Anthropogenic Nutrient Stress
Source: Front Plant Sci. 2021 Jul 5;12:662682. doi: 10.3389/fpls.2021.662682 (PMC8287906; doi:10.3389/fpls.2021.662682)
Supplement: Supplementary file 4 [file Table_1.docx]

**Table S1.** Environmental quality parameters in the seawater and porewater. Inorganic nutrients are expressed in µmol L^-1^ and water parameters (Chl*a*, Corg, C, N and C:N) are reported in µg l^-1^.

n.a: not available. Some parameters could not be measured. For explanation see the Material and Method section.

* Obtained values were below the detection limit.

| Variable | n | June 2019 | | | | September 2019 | | | |  |  |  |
| --- | --- | --- | --- | --- | --- | --- | --- | --- | --- | --- | --- | --- |
|  |  | **S1 Baia** | **S2 Baia** | **S3 Ischia** | **S4 Ischia** | **S1 Baia** | **S2 Baia** | **S3 Ischia** | **S4 Ischia** | | | |
| Seawater | |  | | | |  | | | |  |  |  |
| DIN | 6 | 3.5 ± 0.2 | 4.2 ± 0.1 | 3.9 ± 0.5 | 2.5 ± 0.3 | 0.3 ± 0.0 | 0.3 ± 0.0 | 0.6 ± 0.1 | 0.5 ± 0.1 | | | |
| NH_4_^+^ | 6 | 3.4 ± 0.2 | 3.9 ± 0.2 | 3.3 ± 0.5 | 2.4 ± 0.2 | 0.3 ± 0.0 | 0.3 ± 0.0 | 0.6 ± 0.1 | 0.5 ± 0.1 | | | |
| NO_X_ | 6 | 0.2 ± 0.0 | 0.3 ± 0.1 | 0.5 ± 0.1 | 0.2 ± 0.1 | 0.0 ± 0.0 | 0.0 ± 0.0 | 0.0 ± 0.0 | 0.0 ± 0.0 | | | |
| NO_2_ | 6 | 0.4 ± 0.1 | 0.4 ± 0.2 | 0.6 ± 0.1 | 0.3 ± 0.0 | 0.0 ± 0.0 | 0.0 ± 0.0 | 0.0 ± 0.0 | 0.0 ± 0.0 | | | |
| PO_4_^3-^ | 6 | 2.0 ± 0.3 | 0.5 ± 0.2 | 2.6 ± 0.7 | 0.5 ± 0.2 | 0.1 ± 0.0 | 0.1 ± 0.0 | 0.1 ± 0.0 | 0.1 ± 0.0 | | | |
| Chl*a* | 6 | 0.7 ± 0.1 | 1.4 ± 0.2 | 0.4 ± 0.0 | 0.9 ± 0.2 | n.a | n.a | n.a | n.a | | | |
| Corg | 4 | n.a | n.a | n.a | n.a | 427.76 ± 46.12 | 528.60 ± 8.36 | 0.0 ± 0.0* | 301.61 ± 56.95 | | | |
| C | 4 | n.a | n.a | n.a | n.a | 647.41 ± 129.29 | 717.86 ± 11.94 | 0.0 ± 0.0* | 462.64 ± 81.32 | | | |
| N | 4 | n.a | n.a | n.a | n.a | 84.04 ± 6.26 | 84.58 ± 6.26 | 0.0 ± 0.0* | 50.97 ±9.26 | | | |
| C:N ratio | 4 | n.a | n.a | n.a | n.a | 7.69 ± 0.03 | 8.61 ± 0.84 | 0.0 ± 0.0* | 9.12 ± 0.23 | | | |
| Salinity | 1 | 37.6 | 37.7 | 37.9 | 37.8 | 37.8 | 37.8 | 38.4 | 38.5 | | | |
| pH | 1 | 8.13 | 8.15 | 8.18 | 8.17 | 8.17 | 8.17 | 8.12 | 8.14 | | | |
| Porewater |  |  |  |  |  |  |  |  |  | | | |
| DIN | 6 | n.a | n.a | 12.5 ± 1.8 | 9.8 ± 1.7 | 47.9 ± 4.4 | 31.3 ± 5.7 | 26.7 ± 8.9 | 10.3 ± 4.3 | | | |
| NH_4_^+^ | 6 | n.a | n.a | 10.6 ± 1.9 | 7.5 ± 1.7 | 44.1 ± 4.2 | 29.6 ± 5.9 | 22.6 ± 7.6 | 8.1 ± 3.4 | | | |
| NO_X_ | 6 | n.a | n.a | 1.9 ± 0.5 | 2.3 ± 1.1 | 3.9 ± 0.7 | 1.7 ± 0.5 | 4.1 ± 1.5 | 2.2 ± 1.0 | | | |
| NO_2_ | 6 | n.a | n.a | 0.7 ± 0.2 | 1.2 ± 0.3 | 0.5 ± 0.1 | 0.2 ± 0.1 | 0.6 ± 0.4 | 0.5 ± 0.2 | | | |
| PO_4_^3-^ | 6 | n.a | n.a | 3.4 ± 0.3 | 1.6 ± 0.2 | 4.3 ± 1.0 | 2.6 ± 0.6 | 2.1 ± 0.4 | 1.7 ± 0.1 | | | |
