# Supplementary material for: Unusually Warm Summer Temperatures Exacerbate Population and Plant Level Response of Posidonia oceanica to Anthropogenic Nutrient Stress
Source: Front Plant Sci. 2021 Jul 5;12:662682. doi: 10.3389/fpls.2021.662682 (PMC8287906; doi:10.3389/fpls.2021.662682)
Supplement: Supplementary file 5 [file Table_2.docx]

**Table S2.** Results of the Kruskal-Wallis rank sum tests for the different water parameters with paired t-test comparison between sites that receive different levels of anthropogenic pressures.

| Kruskal-Wallis rank sum test | **Chla ~ condition** |  |  |  |
| --- | --- | --- | --- | --- |
|  |  | chi-squared | df | p |
|  |  | 4.864 | 1 | **0.0274** |
|  |  |  |  |  |
| Pairwise Wilcoxon rank sum test |  |  |  |  |
|  | impacted |  |  |  |
| pristine | **0.028** |  |  |  |

|  | **Corg ~ condition** |  |  |  |
| --- | --- | --- | --- | --- |
|  |  | chi-squared | df | p |
|  |  | 8.2479 | 1 | **0.0041** |
|  |  |  |  |  |
| Pairwise Wilcoxon rank sum test |  |  |  |  |
|  | impacted |  |  |  |
| pristine | **0.005** |  |  |  |

|  | **N ~ condition** |  |  |  |
| --- | --- | --- | --- | --- |
|  |  | chi-squared | df | p |
|  |  | 7.5152 | 1 | **0.0061** |
|  |  |  |  |  |
| Pairwise Wilcoxon rank sum test |  |  |  |  |
|  | impacted |  |  |  |
| pristine | **0.0074** |  |  |  |

|  | **C ~ condition** |  |  |  |
| --- | --- | --- | --- | --- |
|  |  | chi-squared | df | p |
|  |  | 8.2479 | 1 | **0.0041** |
|  |  |  |  |  |
| Pairwise Wilcoxon rank sum test |  |  |  |  |
|  | impacted |  |  |  |
| pristine | **0.005** |  |  |  |

|  | **CN ratio ~ condition** |  |  |  |
| --- | --- | --- | --- | --- |
|  |  | chi-squared | df | p |
|  |  | 2.7266 | 1 | 0.6016 |
|  |  |  |  |  |
| ---------------------------------------- |  |  |  |  |
