# Supplementary material for: Unusually Warm Summer Temperatures Exacerbate Population and Plant Level Response of Posidonia oceanica to Anthropogenic Nutrient Stress
Source: Front Plant Sci. 2021 Jul 5;12:662682. doi: 10.3389/fpls.2021.662682 (PMC8287906; doi:10.3389/fpls.2021.662682)
Supplement: Supplementary file 6 [file Table_3.docx]

**Table S3.** Data for the four subsites on all measured traits (population level morphological and biochemical).

| Traits | Site | June 2019 | | September 2019 | |
| --- | --- | --- | --- | --- | --- |
|  |  | **Control** | **Fertilized** | **Control** | **Fertilized** |
| Population level traits | | | | | |
| Cover [%] | S1- Baia | 50.00 ± 13.23 | 53.33 ± 10.14 | 26.67 ± 1.67 | 18.33 ± 6.01 |
|  | S2- Baia | 73.33 ± 4.41 | 56.67 ± 3.33 | 30.00 ± 2.89 | 20.00 ± 2.89 |
|  | S3- Ischia | 85.00 ± 2.89 | 88.33 ± 6.01 | 88.33 ± 4.41 | 68.33 ± 4.41 |
|  | S4- Ischia | 78.33 ± 4.41 | 86.67 ± 6.01 | 71.67 ± 4.41 | 63.33 ± 1.67 |
| Shoot density [shoots m^-2^] | S1- Baia | 133.33 ± 24.00 | 112.00 ± 23.70 | 129.78 ± 18.17 | 131.56 ± 5.83 |
|  | S2- Baia | 112.00 ± 17.28 | 188.44 ± 31.41 | 188.44 ± 31.41 | 170.67 ± 21.99 |
|  | S3- Ischia | 288.00 ± 25.99 | 268.44 ± 25.27 | 227.56 ± 19.00 | 250.67 ± 34.46 |
|  | S4- Ischia | 229.33 ± 37.62 | 248.89 ± 27.21 | 261.33 ± 14.85 | 245.33 ± 32.66 |
| Epiphyte cover [mg (DW) cm^-2^ shoot^-1^] | S1- Baia | 5.38 ± 0.49 | 5.55 ± 0.58 | 4.41 ± 0.69 | 11.87 ± 1.87 |
|  | S2- Baia | 3.46 ± 0.40 | 3.51 ± 0.23 | 3.46 ± 0.24 | 5.01 ± 0.85 |
|  | S3- Ischia | 0.62 ± 0.12 | 0.91 ± 0.17 | 1.84 ± 0.12 | 1.43 ± 0.30 |
|  | S4- Ischia | 1.80 ± 0.65 | 0.95 ± 0.15 | 1.92 ± 0.34 | 1.96 ± 0.42 |
| Morphological traits | | | | | |
| No. leaves per shoot | S1- Baia | 5.00 ± 0.26 | 5.78 ± 0.22 | 3.44 ± 0.24 | 3.89 ± 0.39 |
|  | S2- Baia | 5.89 ± 0.20 | 5.56 ± 0.12 | 3.89 ± 0.26 | 3.33 ± 0.24 |
|  | S3- Ischia | 4.33 ± 0.41 | 4.56 ± 0.12 | 4.44 ± 0.24 | 4.33 ± 0.29 |
|  | S4- Ischia | 5.00 ± 0.29 | 4.56 ± 0.30 | 5.00 ± 0.33 | 4.11 ± 0.20 |
| Leaf height [cm] | S1- Baia | 58.71 ± 3.15 | 55.14 ± 3.62 | 25.48 ± 3.74 | 24.70 ± 3.35 |
|  | S2- Baia | 52.74 ± 3.10 | 53.80 ± 3.45 | 28.37 ± 3.86 | 30.56 ± 4.56 |
|  | S3- Ischia | 72.75 ± 5.22 | 72.05 ± 4.25 | 43.55 ± 5.60 | 36.92 ± 4.62 |
|  | S4- Ischia | 67.48 ± 4.44 | 70.06 ± 3.80 | 42.83 ± 4.88 | 37.26 ± 4.99 |
| Max. leaf canopy height [mm] | S1- Baia | 822.22 ± 30.27 | 878.89 ± 60.65 | 449.00 ± 56.32 | 466.67 ± 43.23 |
|  | S2- Baia | 782.78 ± 21.17 | 816.44 ± 35.84 | 531.33 ± 43.02 | 558.67 ± 49.70 |
|  | S3- Ischia | 1001.11 ± 74.30 | 982.22 ± 50.26 | 831.89 ± 40.23 | 708.56 ± 51.24 |
|  | S4- Ischia | 958.33 ± 60.65 | 903.89 ± 44.88 | 817.56 ± 52.55 | 680.33 ± 54.42 |
| LAI [m² leaf area per shoot * shoot density per m²] | S1- Baia | 3.17 ± 0.39 | 3.07 ± 0.28 | 1.20 ± 0.23 | 1.51 ± 0.15 |
|  | S2- Baia | 2.68 ± 0.39 | 4.60 ± 0.57 | 1.74 ± 0.28 | 1.96 ± 0.22 |
|  | S3- Ischia | 9.01 ± 1.24 | 8.38 ± 0.71 | 3.77 ± 0.48 | 3.58 ± 0.33 |
|  | S4- Ischia | 5.80 ± 0.86 | 6.56 ± 0.52 | 3.82 ± 0.57 | 3.13 ± 0.49 |
| Leaf width [cm] | S1- Baia | 1.00 ± 0.00 | 1.00 ± 0.00 | 1.09 ± 0.04 | 1.11 ± 0.02 |
|  | S2- Baia | 1.00 ± 0.00 | 1.00 ± 0.00 | 1.11 ± 0.03 | 1.13 ± 0.03 |
|  | S3- Ischia | 0.96 ± 0.02 | 0.94 ± 0.02 | 1.01 ± 0.03 | 1.04 ± 0.03 |
|  | S4- Ischia | 0.87 ± 0.03 | 0.88 ± 0.02 | 0.94 ± 0.02 | 0.90 ± 0.02 |
| Leaf area [cm] | S1- Baia | 58.71 ± 3.19 | 55.14 ± 3.77 | 27.85 ± 4.18 | 27.95 ± 3.89 |
|  | S2- Baia | 52.74 ± 3.13 | 53.80 ± 3.38 | 31.66 ± 4.41 | 34.83 ± 5.26 |
|  | S3- Ischia | 70.11 ± 5.26 | 68.47 ± 4.13 | 44.43 ± 5.52 | 36.92 ± 4.62 |
|  | S4- Ischia | 59.11 ± 4.32 | 61.59 ± 3.46 | 40.52 ± 4.62 | 33.69 ± 4.55 |
| Leaf area per shoot [cm] | S1- Baia | 234.44 ± 16.82 | 251.11 ± 15.51 | 92.70 ± 17.77 | 99.97 ± 16.80 |
|  | S2- Baia | 232.70 ± 20.56 | 239.48 ± 17.94 | 110.75 ± 16.75 | 116.09 ± 13.78 |
|  | S3- Ischia | 303.79 ± 29.85 | 311.88 ± 24.92 | 162.81 ± 17.07 | 147.06 ± 13.42 |
|  | S4- Ischia | 247.70 ± 31.32 | 261.84 ± 18.15 | 145.26 ± 20.10 | 119.82 ± 16.51 |
| Biochemical traits | | | | | |
| Leaf C [% DW] | S1- Baia | 31.20 ± 0.62 | 31.51 ± 0.30 | 34.06 ± 0.13 | 34.32 ± 0.63 |
|  | S2- Baia | 31.38 ± 0.88 | 32.76 ± 0.13 | 35.75 ± 1.39 | 35.77 ± 0.70 |
|  | S3- Ischia | 31.27 ± 0.21 | 31.45 ± 0.32 | 33.73 ± 0.06 | 35.60 ± 0.40 |
|  | S4- Ischia | 32.50 ± 0.12 | 32.25 ± 0.15 | 33.90 ± 0.90 | 33.91 ± 0.16 |
| Rhizome C [% DW] | S1- Baia | 38.50 ± 1.31 | 39.50 ± 0.24 | 40.67 ± 0.51 | 39.54 ± 3.08 |
|  | S2- Baia | 40.35 ± 0.33 | 40.63 ± 0.37 | 43.82 ± 2.82 | 40.02 ± 0.36 |
|  | S3- Ischia | 40.61 ± 0.45 | 39.46 ± 0.30 | 40.88 ± 0.67 | 41.22 ± 0.34 |
|  | S4- Ischia | 39.14 ± 0.40 | 40.16 ± 0.07 | 41.37 ± 0.48 | 43.62 ± 3.19 |
| Leaf N [% DW] | S1- Baia | 2.00 ± 0.07 | 1.82 ± 0.15 | 2.11 ± 0.25 | 2.35 ± 0.31 |
|  | S2- Baia | 1.64 ± 0.08 | 1.74 ± 0.16 | 2.07 ± 0.50 | 2.02 ± 0.23 |
|  | S3- Ischia | 0.82 ± 0.02 | 1.13 ± 0.09 | 0.94 ± 0.08 | 1.40 ± 0.13 |
|  | S4- Ischia | 1.43 ± 0.09 | 1.35 ± 0.16 | 1.11 ± 0.20 | 1.21 ± 0.16 |
| Rhizome N [% DW] | S1- Baia | 3.10 ± 0.88 | 3.37 ± 0.84 | 5.52 ± 0.65 | 3.91 ± 0.36 |
|  | S2- Baia | 2.79 ± 0.67 | 3.03 ± 0.29 | 4.61 ± 0.31 | 3.48 ± 0.81 |
|  | S3- Ischia | 1.53 ± 0.29 | 2.01 ± 0.43 | 1.99 ± 0.21 | 2.47 ± 0.29 |
|  | S4- Ischia | 1.20± 0.36 | 1.80 ± 0.16 | 2.54 ± 0.21 | 4.05 ± 0.32 |
| Leaf δ13C [‰] | S1- Baia | -12.77 ± 0.26 | -12.40 ± 0.42 | -14.58 ± 0.85 | -13.48 ± 0.63 |
|  | S2- Baia | -12.85 ± 0.13 | -12.87 ± 0.70 | -13.63 ± 0.46 | -13.30 ± 0.47 |
|  | S3- Ischia | -12.46 ± 0.08 | -12.96 ± 0.24 | -12.61 ± 0.82 | -13.08 ± 0.45 |
|  | S4- Ischia | -13.48 ± 0.32 | -13.87 ± 0.86 | -11.94 ± 0.78 | -12.81 ± 0.49 |
| Rhizome δ13C [‰] | S1- Baia | -13.57 ± 0.46 | -12.87 ± 0.12 | -13.23 ± 0.23 | -13.04 ± 0.35 |
|  | S2- Baia | -12.84 ± 0.23 | -12.33 ± 0.07 | -13.07 ± 0.03 | -12.83 ± 0.35 |
|  | S3- Ischia | -13.33 ± 0.15 | -13.21 ± 0.20 | -12.46 ± 0.37 | -13.08 ± 0.19 |
|  | S4- Ischia | -13.33 ± 0.23 | -13.30 ± 0.57 | -12.96 ± 0.23 | -13.14 ± 0.19 |
| Leaf δ15N [‰] | S1- Baia | 6.73 ± 0.63 | 7.20 ± 0.18 | 5.32 ± 0.11 | 5.10 ± 0.11 |
|  | S2- Baia | 5.94 ± 0.23 | 6.27 ± 0.28 | 6.14 ± 0.44 | 4.71 ± 0.32 |
|  | S3- Ischia | 4.66 ± 0.84 | 7.14 ± 0.49 | 5.37 ± 0.20 | 4.15 ± 0.46 |
|  | S4- Ischia | 6.29 ± 0.19 | 6.41 ± 0.17 | 4.22 ± 0.99 | 4.19 ± 0.62 |
| Rhizome δ15N [‰] | S1- Baia | 6.72 ± 0.05 | 6.64 ± 0.42 | 6.32 ± 0.06 | 6.18 ± 0.12 |
|  | S2- Baia | 6.23 ± 0.41 | 6.74 ± 0.26 | 6.09 ± 0.08 | 5.51 ± 0.34 |
|  | S3- Ischia | 6.43 ± 0.32 | 6.72 ± 0.55 | 5.65 ± 0.10 | 5.18 ± 0.30 |
|  | S4- Ischia | 5.37 ± 0.37 | 5.51 ± 0.12 | 5.09 ± 0.08 | 5.32 ± 0.47 |
| Leaf C:N ratio | S1- Baia | 15.90 ± 0.82 | 17.60 ± 1.62 | 16.56 ± 1.84 | 15.19 ± 2.98 |
|  | S2- Baia | 19.30 ± 1.48 | 19.14 ± 1.53 | 18.99 ± 3.56 | 18.13 ± 1.95 |
|  | S3- Ischia | 38.17 ± 1.13 | 28.30 ± 2.09 | 36.50 ± 3.21 | 25.90 ± 2.35 |
|  | S4- Ischia | 22.89 ± 1.46 | 24.49 ± 2.77 | 32.33 ± 5.03 | 28.96 ± 3.82 |
| Rhizome C:N ratio | S1- Baia | 14.60 ± 4.49 | 12.86 ± 4.47 | 7.58 ± 0.89 | 10.15 ± 1.23 |
|  | S2- Baia | 16.39 ± 4.12 | 13.61 ± 1.08 | 9.69 ± 1.33 | 12.82 ± 2.85 |
|  | S3- Ischia | 28.52 ± 5.13 | 21.26 ± 3.90 | 20.99 ± 2.13 | 17.20 ± 2.03 |
|  | S4- Ischia | 40.37 ± 13.55 | 22.71 ± 2.01 | 16.56 ± 1.47 | 10.91 ± 1.09 |
| Leaf P [µg g^-1^] | S1- Baia | 886.33 ± 70.44 | 1083.00 ± 299.96 | 491.02 ± 116.48 | 489.61 ± 152.98 |
|  | S2- Baia | 1161.67 ± 408.41 | 1022.00 ± 272.86 | 1318.67 ± 529.22 | 964.36 ± 371.55 |
|  | S3- Ischia | 508.33 ± 14.84 | 579.00 ± 19.98 | 621.67 ± 101.64 | 710.00 ± 70.50 |
|  | S4- Ischia | 675.00 ± 40.87 | 710.00 ± 145.34 | 686.33 ± 112.79 | 735.67 ± 131.82 |
| Rhizome P [µg g^-1^] | S1- Baia | 311.67 ± 53.17 | 726.67 ± 299.54 | 1373.33 ± 210.98 | 1177.33 ± 422.91 |
|  | S2- Baia | 561.00 ± 52.94 | 988.67 ± 337.05 | 2208.00 ± 145.94 | 1815.33 ± 292.31 |
|  | S3- Ischia | 426.33 ± 122.38 | 665.33 ± 177.20 | 1193.33 ± 105.44 | 1492.67 ± 129.91 |
|  | S4- Ischia | 599.67 ± 258.14 | 596.00 ± 36.77 | 1173.00 ± 129.81 | 1879.00 ± 385.72 |
| Starch in the rhizome [sucrose eq mg^−1^ DW] | S1- Baia | 72.73 ± 8.08 | 115.70 ± 27.16 | 88.55 ± 9.32 | 77.32 ± 11.78 |
|  | S2- Baia | 122.13 ± 13.99 | 134.20 ± 41.20 | 127.89 ± 22.54 | 105.17 ± 12.70 |
|  | S3- Ischia | 153.37 ± 14.03 | 123.36 ± 16.00 | 131.79 ± 5.69 | 179.69 ± 26.05 |
|  | S4- Ischia | 109.87 ± 10.88 | 118.76 ± 13.40 | 198.37 ± 35.01 | 126.84 ± 7.54 |
| Sugar in the rhizome [sucrose eq mg^−1^ DW] | S1- Baia | 175.30 ± 46.38 | 187.25 ± 8.24 | 143.85 ± 18.98 | 189.25 ± 7.20 |
|  | S2- Baia | 192.00 ± 16.66 | 171.15 ± 23.87 | 146.72 ± 12.72 | 173.77 ± 4.98 |
|  | S3- Ischia | 232.98 ± 17.61 | 237.59 ± 17.15 | 198.66 ± 23.36 | 194.01 ± 35.91 |
|  | S4- Ischia | 262.11 ± 48.72 | 212.73 ± 10.40 | 233.59 ± 24.77 | 212.03 ± 49.85 |
| Starch in the leaf [sucrose eq mg^−1^ DW] | S1- Baia | 69.04 ± 19.77 | 35.45 ± 6.92 | 46.24 ± 4.28 | 71.43 ± 2.13 |
|  | S2- Baia | 54.01 ± 4.81 | 38.98 ± 4.12 | 27.38 ± 8.67 | 45.47 ± 6.86 |
|  | S3- Ischia | 59.49 ± 4.18 | 68.12 ± 6.91 | 58.88 ± 9.12 | 54.26 ± 1.33 |
|  | S4- Ischia | 72.86 ± 4.47 | 65.14 ± 11.15 | 55.97 ± 6.38 | 44.23 ± 3.75 |
| Sugar in the leaf [sucrose eq mg^−1^ DW] | S1- Baia | 20.46 ± 1.44 | 19.78 ± 14.46 | 29.41 ± 3.16 | 30.15 ± 4.78 |
|  | S2- Baia | 27.52 ± 4.90 | 39.24 ± 11.45 | 25.07 ± 3.56 | 30.04 ± 2.97 |
|  | S3- Ischia | 43.86 ± 5.41 | 44.68 ± 0.74 | 48.61 ± 9.77 | 44.32 ± 4.39 |
|  | S4- Ischia | 16.41 ± 3.12 | 31.26 ± 8.12 | 30.84 ± 4.58 | 39.04 ± 11.64 |
