# Supplementary material for: Unusually Warm Summer Temperatures Exacerbate Population and Plant Level Response of Posidonia oceanica to Anthropogenic Nutrient Stress
Source: Front Plant Sci. 2021 Jul 5;12:662682. doi: 10.3389/fpls.2021.662682 (PMC8287906; doi:10.3389/fpls.2021.662682)
Supplement: Supplementary file 8 [file Table_5.docx]

**Table S5.** Linear mixed effect model (LME) selection for population level responses of *P. oceanica* over time (June 2019 to September 2019). df = degrees of freedom. AICc = Akaike Information Criterion corrected for small sample sizes. ΔAICc = difference AICc values between each model and the best fitting model with the lowest AICc. AICcWt = Akaike weights. LL= Likelihood. The significance of time was assessed using the likelihood ratio (LR) test by comparing models with the time added against the null model.

| Model ranking | Model | df | AICc | ΔAICc | AICcWt | LL | χ2 | p value | R² |
| --- | --- | --- | --- | --- | --- | --- | --- | --- | --- |
| Cover | | | | | | | | | |
| **1** | **Cover ~ time** | **4** | **212.3** | **0.0** | **0.940** | **-101.10** | **8.42** | **0.0037** | **0.748** |
| 2 | Intercept only (Cover ~ 1) | 3 | 217.8 | 5.5 | 0.060 | -105.30 |  |  |  |
| Shoot density | | | | | | | | | |
| 1 | Sdens ~ time | 4 | 269. 7 | 0 | 0.754 | -131.24 | 0.67 | 0.4133 | 0.623 |
| 2 | Intercept only (Sdens ~ 1) | 3 | 271.9 | 2.2 | 0.246 | -130.90 |  |  |  |
| Epiphytes | | | | | | | | | |
| 1 | Intercept only (Epi ~ 1) | 3 | 76.1 | 0.0 | 0.8040 | -34.45 | 0.77 | 0.0822 | 0.796 |
| 2 | Epi ~ time | 4 | 78.9 | 2.8 | 0.1960 | -34.41 |  |  |  |
