# Supplementary material for: Unusually Warm Summer Temperatures Exacerbate Population and Plant Level Response of Posidonia oceanica to Anthropogenic Nutrient Stress
Source: Front Plant Sci. 2021 Jul 5;12:662682. doi: 10.3389/fpls.2021.662682 (PMC8287906; doi:10.3389/fpls.2021.662682)
Supplement: Supplementary file 10 [file Table_7.docx]

**Table S7.** Linear mixed effect model (LME) selection for morphological responses of *P. oceanica* over time (June 2019 to September 2019). df = degrees of freedom. AICc = Akaike Information Criterion corrected for small sample sizes. ΔAICc = difference AICc values between each model and the best fitting model with the lowest AICc. AICcWt = Akaike weights. LL= Likelihood. The significance of time was assessed using the likelihood ratio (LR) test by comparing models with the time added against the null model.

| Model ranking | Model | df | AICc | ΔAICc | AICcWt | LL | χ2 | p value | R² |
| --- | --- | --- | --- | --- | --- | --- | --- | --- | --- |
| Number of leaves per shoot | | | | | | | | | |
| **1** | **Number ~ time** | **4** | **209.1** | **0.0** | **0.996** | **-100.24** | **13.29** | **0.0003** | **0.236** |
| 2 | Intercept only (Number ~ 1) | 3 | 220.1 | 11.1 | 0.004 | -106.88 |  |  |  |
| Leaf length | | | | | | | | | |
| **1** | **height ~ time** | **4** | **3173.9** | **0.0** | **1.000** | **-1582.88** | **69.77** | **<0.0001** | **0.228** |
| 2 | Intercept only (height ~ 1) | 3 | 3241.6 | 67.7 | 0.000 | -1617.77 |  |  |  |
| Maximum leaf canopy height | | | | | | | | | |
| **1** | **c_height ~ time** | **4** | **615.6** | **0.0** | **1.000** | **-303.50** | **32.82** | **<0.0001** | **0.577** |
| 2 | Intercept only (c_height ~ 1) | 3 | 646.2 | 30.6 | 0.000 | -319.90 |  |  |  |
| Leaf width | | | | | | | | | |
| **1** | **width ~ time** | **4** | **-157.9** | **0.0** | **1.000** | **83.24** | **21.79** | **<0.0001** | **0.532** |
| 2 | Intercept only (width ~ 1) | 3 | -138.3 | 19.6 | 0.000 | 72.34 |  |  |  |
| Leaf area | | | | | | | | | |
| **1** | **area ~ time** | **4** | **3169.6** | **0.0** | **1.000** | **-1580.47** | **53.60** | **<0.0001** | **0.174** |
| 2 | Intercept only (area ~ 1) | 3 | 3220.6 | 51.6 | 0.000 | -1607.27 |  |  |  |
| Leaf area per shoot | | | | | | | | | |
| **1** | **l_area ~ time** | **4** | **817.6** | **0.0** | **1.000** | **-40.45** | **47.86** | **<0.0001** | **0.540** |
| 2 | Intercept only (l_area ~ 1) | 3 | 863.3 | 45.6 | 0.000 | -42.85 |  |  |  |
| LAI | | | | | | | | | |
| **1** | **LAI ~ time** | **4** | **326.1** | **0.0** | **1.000** | **-158.77** | **23.26** | **<0.0001** | **0.550** |
| 2 | Intercept only (LAI ~ 1) | 3 | 347.2 | 2.1 | 0.000 | -170.40 |  |  |  |
