# Supplementary material for: Unusually Warm Summer Temperatures Exacerbate Population and Plant Level Response of Posidonia oceanica to Anthropogenic Nutrient Stress
Source: Front Plant Sci. 2021 Jul 5;12:662682. doi: 10.3389/fpls.2021.662682 (PMC8287906; doi:10.3389/fpls.2021.662682)
Supplement: Supplementary file 12 [file Table_9.docx]

**Table S9.** Linear mixed effect model (LME) selection for nutrient content in leaves and rhizomes of *P. oceanica* over time (June 2019 to September 2019). df = degrees of freedom. AICc = Akaike Information Criterion corrected for small sample sizes. ΔAICc = difference AICc values between each model and the best fitting model with the lowest AICc. AICcWt = Akaike weights. LL= Likelihood. The significance of time was assessed using the likelihood ratio (LR) test by comparing models with the time added against the null model.

| Model ranking | Model | df | AICc | ΔAICc | AICcWt | LL | χ2 | p value | R² |
| --- | --- | --- | --- | --- | --- | --- | --- | --- | --- |
| CN ratio- Leaves | | | | | | | | | |
| 1 | Intercept only (cnleaves ~ 1) | 3 | 163.2 | 0.0 | 0.716 | -78.02 | 1.06 | 0.3033 | 0.768 |
| 2 | cnleaves ~ time | 4 | 165.1 | 1.9 | 0.284 | -77.49 |  |  |  |
| Carbon content - Leaves | | | | | | | | | |
| **1** | **C ~ time** | **4** | **87.4** | **0.0** | **1.000** | **-38.63** | **20.05** | **<0.0001** | **0.566** |
| 2 | Intercept only (C ~ 1) | 3 | 104.5 | 17.1 | 0.000 | -48.66 |  |  |  |
| Nitrogen content - Leaves | | | | | | | | | |
| 1 | Intercept only (N ~ 1) | 3 | 36.5 | 0.0 | 0.780 | -14.67 | 0.37 | 0.5454 | 0.653 |
| 2 | N ~ time | 4 | 39.1 | 2.5 | 0.220 | -14.49 |  |  |  |
| Phosphorous content - Leaves | | | | | | | | | |
| **1** | **P ~ time** | **4** | **366.9** | **2.8** | **0.808** | **-179.85** | **0.86** | **0.030** | **0.338** |
| 2 | Intercept only (P ~ 1) | 3 | 369.8 | 0.0 | 0.190 | -179.83 |  |  |  |
| CN ratio- Rhizomes | | | | | | | | | |
| **1** | **cnrhizomes~ time** | **4** | **192.0** | **0.0** | **0.880** | **-90.95** | **6.89** | **0.0087** | **0.514** |
| 2 | Intercept only (cnrhizomes ~ 1) | 3 | 196.0 | 4.0 | 0.120 | -94.39 |  |  |  |
| Carbon content- Rhizomes | | | | | | | | | |
| **1** | **C ~ time** | **4** | **110.8** | **0.0** | **0.808** | **-50.37** | **47.86** | **<0.0001** | **0.288** |
| 2 | Intercept only (C ~ 1) | 3 | 113.7 | 2.9 | 0.192 | -53.26 |  |  |  |
| Nitrogen content- Rhizomes | | | | | | | | | |
| **1** | **N~ time** | **4** | **80.8** | **0.0** | **0.993** | **-35.34** | **12.80** | **0.0001** | **0.740** |
| 2 | Intercept only (N~ 1) | 3 | 90.6 | 9.8 | 0.007 | -41.69 |  |  |  |
| Phosphorous content- Rhizomes | | | | | | | | | |
| **1** | **P ~ time** | **4** | **360.0** | **0.0** | **1.000** | **-174.96** | **27.63** | **<0.0001** | **0.770** |
| 2 | Intercept only (P ~ 1) | 3 | 384.7 | 24.7 | 0.000 | -188.77 |  |  |  |
