# Supplementary material for: Unusually Warm Summer Temperatures Exacerbate Population and Plant Level Response of Posidonia oceanica to Anthropogenic Nutrient Stress
Source: Front Plant Sci. 2021 Jul 5;12:662682. doi: 10.3389/fpls.2021.662682 (PMC8287906; doi:10.3389/fpls.2021.662682)
Supplement: Supplementary file 14 [file Table_11.docx]

**Table S11.** Linear mixed effect model (LME) selection for natural stable isotope content (δ^15^N and δ^13^C) in leaves and rhizomes of *P. oceanica* over time (June 2019 to September 2019). df = degrees of freedom. AICc = Akaike Information Criterion corrected for small sample sizes. ΔAICc = difference AICc values between each model and the best fitting model with the lowest AICc. AICcWt = Akaike weights. LL= Likelihood. The significance of time was assessed using the likelihood ratio (LR) test by comparing models with the time added against the null model.

| Model ranking | Model | df | AICc | ΔAICc | AICcWt | LL | χ2 | p value | R² |
| --- | --- | --- | --- | --- | --- | --- | --- | --- | --- |
| δ^13^C – Leaves | | | | | | | | | |
| 1 | Intercept only (13C ~ 1) | 3 | 79.6 | 0.0 | 0.773 | -36.20 | 0.46 | 0.4993 | 0.168 |
| 2 | 13C ~ time | 4 | 82.0 | 2.4 | 0.227 | -35.97 |  |  |  |
| δ^15^N - Leaves | | | | | | | | | |
| 1 | Intercept only (15N ~ 1) | 3 | 80.5 | 0.0 | 0.597 | -36.64 | 2.12 | 0.1452 | 0.168 |
| 2 | 15N ~ time | 4 | 81.3 | 0.8 | 0.403 | -35.58 |  |  |  |
| δ^13^C - Rhizomes | | | | | | | | | |
| 1 | Intercept only (13C ~ 1) | 3 | 42.1 | 0.0 | 0.505 | -17.46 | 2.87 | 0.0905 | 0.154 |
| 2 | 13C ~ time | 4 | 42.2 | 0.0 | 0.495 | -16.03 |  |  |  |
| δ^15^N - Rhizomes | | | | | | | | | |
| **1** | **15N ~ time** | **4** | **40.8** | **0.0** | **0.796** | **-15.33** | **5.63** | **0.0177** | **0.683** |
| 2 | Intercept only (15N ~ 1) | 3 | 43.5 | 2.7 | 0.204 | -18.15 |  |  |  |
