# Supplementary material for: Unusually Warm Summer Temperatures Exacerbate Population and Plant Level Response of Posidonia oceanica to Anthropogenic Nutrient Stress
Source: Front Plant Sci. 2021 Jul 5;12:662682. doi: 10.3389/fpls.2021.662682 (PMC8287906; doi:10.3389/fpls.2021.662682)
Supplement: Supplementary file 16 [file Table_13.docx]

**Table S13.** Linear mixed effect model (LME) selection for nonstructural carbohydrate concentrations in leaves and rhizomes of *P. oceanica* over time (June 2019 to September 2019). df = degrees of freedom. AICc = Akaike Information Criterion corrected for small sample sizes. ΔAICc = difference AICc values between each model and the best fitting model with the lowest AICc. AICcWt = Akaike weights. LL= Likelihood. The significance of time was assessed using the likelihood ratio (LR) test by comparing models with the time added against the null model.

| Model ranking | Model | df | AICc | ΔAICc | AICcWt | LL | χ2 | p value | R² |
| --- | --- | --- | --- | --- | --- | --- | --- | --- | --- |
| Sugar – Leaves | | | | | | | | | |
| 1 | sugar ~ time | 4 | 188.9 | 0.0 | 0.529 | -89.40 | 3.14 | 0.764 | 0.612 |
| 2 | Intercept only (sugar ~ 1) | 3 | 189.1 | 0.2 | 0.471 | -90.97 |  |  |  |
| Starch - Leaves | | | | | | | | | |
| **1** | **starch ~ time** | **4** | **211.6** | **0.0** | **0.840** | **-100.77** | **6.22** | **0.0126** | **0.396** |
| 2 | Intercept only (starch ~ 1) | 3 | 215.0 | 3.3 | 0.160 | -103.88 |  |  |  |
| Sugar - Rhizomes | | | | | | | | | |
| 1 | sugar ~ time | 4 | 266.5 | 0.0 | 0.547 | -128.20 | 3.28 | 0.0702 | 0.472 |
| 2 | Intercept only (sugar ~ 1) | 3 | 266.9 | 0.4 | 0.453 | -129.84 |  |  |  |
| Starch - Rhizomes | | | | | | | | | |
| 1 | Intercept only (starch ~ 1) | 3 | 253.6 | 0.0 | 0.580 | -123.20 | 2.26 | 0.1324 | 0.406 |
| 2 | starch ~ time | 4 | 254.2 | 0.6 | 0.420 | -122.07 |  |  |  |
